# Data set on sedimentology, palaeoecology and chronology of Middle to Late Pleistocene deposits on the Taimyr Peninsula, Arctic Russia

**DOI:** 10.1016/j.dib.2019.104267

**Published:** 2019-07-17

**Authors:** Per Möller, Ívar Örn Benediktsson, Johanna Anjar, Ole Bennike, Martin Bernhardson, Svend Funder, Lena M. Håkansson, Geoffrey Lemdahl, Joseph M. Licciardi, Andrew S. Murray, Marit-Solveig Seidenkrantz

**Affiliations:** a Department of Geology, Quaternary Sciences, Lund University, Sölvegatan 12, SE-223 62 Lund, Sweden; b Institute of Earth Sciences, University of Iceland, Sturlugata 7, IS-101 Reykjavík, Iceland; c Department of Natural Sciences and Environmental Health, University of South-Eastern Norway, Gullbringvegen 36, 3800, Bø, Norway; d GEUS, Øster Voldgade 10, DK-1350 København K, Denmark; e Geological Museum, University of Copenhagen, Øster Voldgade 5-7, DK-1350 København K, Denmark; f The University Centre in Svalbard (UNIS), P.O. Box 156 N-9171 Longyearbyen, Norway; g Department of Biology and Environmental Science, Linnaeus University, SE-39182 Kalmar, Sweden; h Department of Earth Sciences, University of New Hampshire, 56 College Road, Durham, NH, 03824, USA; i The Nordic Laboratory for Luminescence Dating, Department of Earth Sciences, Aarhus University, Risø National Laboratory, DK-4000 Roskilde, Denmark; j Paleoceanography and Paleoclimate Group, Arctic Research Centre, and iClimate, Interdisciplinary Centre for Climate Change, Aarhus University, Høegh Guldbergs Gade 2, DK-8000 Aarhus C, Denmark

**Keywords:** Taimyr, Glacial sedimentology, Glacial history, Kara Sea ice sheet, OSL dating, ESR dating, TCN dating

## Abstract

This Data in Brief paper contains data (including images) from Quaternary sedimentary successions investigated along the Bol'shaya Balakhnya River and the Luktakh–Upper Taimyra–Logata river system on southern Taimyr Peninsula, NW Siberia (Russia). Marine foraminifera and mollusc fauna composition, extracted from sediment samples, is presented. The chronology (time of deposition) of the sediment successions is reconstructed from three dating methods; (i) radiocarbon dating of organic detritus (from lacustrine/fluvial sediment) and molluscs (marine sediment) as finite ages (usually <42 000 years) or as non-finite ages (>42 000–48 000 years) on samples/sediments beyond the radiocarbon dating limit; (ii) Electron Spin Resonance (ESR) dating on marine molluscs (up to ages >400 000 years); (iii) Optically Stimulated Luminescence (OSL) dating, usually effective up to 100–150 0000 years. Terrestrial Cosmogenic Nuclide (TCN) exposure dating has been applied to boulders resting on top of moraine ridges (Ice Marginal Zones). See (Möller et al., 2019) (doi.org/10.1016/j.earscirev.2019.04.004) for interpretation and discussion of all data.

**Table:** Specifications table

Subject area	*Geology*
More specific subject area	*Quaternary palaeo-environmental reconstruction*
Type of data	*Photo documentation of sediment successions. Marine and terrestrial fauna and flora lists from the sediments. Lists of Optically Stimulated Luminescence) (OSL), Electron Spin Resonance (ESR), AMS radiocarbon (*^*14*^*C) and Terrestrial Cosmogenic Nuclide (TCN) exposure ages. Tables and figures.*
How data was acquired	*The logging and photographing of excavated sedimentary successions (see logs in*[1])*, as well as sampling for palaeontological analyses and dating (all sampling points shown in sediment logs in (*[1]), *took place during boat cruises along the Bol'shaya Balaknya River and the Luktakh–Upper Taimyra–Logata river systems on the Taimyr Peninsula, NW Siberia, in 2010 and 2012. Field sampling procedures are described in text, as well as laboratory procedures.*
Data format	*Raw and analysed*
Experimental factors	*Sediment successions in river-cut bluffs and solifluction scars were cleaned in vertical sections close to the permafrost table and logged to their lithofacies (*Table 1*), and sampled for palaeontological analysis (*Table 2, Table 3, Table 4*) and dating (*^*14*^*C, ESR, OSL;*Table 5, Table 6, Table 7*). Erratic boulders on Ice Marginal Zones were sampled for TCN dating (*Table 8, Table 9, Table 10*).*
Experimental features	*Sediment succession logging provide basis for palaeoenvironmental interpretation for discerned sediment units at the specific site and retrieved chronological data (*^*14*^*C, ESR, OSL, TCN ages) form a base for temporal environmental reconstructions on a regional scale.*
Data source location	*Taimyr Peninsula, northwest Siberia, Russia, c. between coordinates N71˚5’ -74˚15′ and E92˚15′-106˚0’ (see*Fig. 1*)*
Data accessibility	*Data is within this article*
Related research article	• Möller, P., Benediktsson, Í.Ö., Anjar, J., Bennike, O., Bernhardson, M., Funder, S., Håkansson, L., Lemdahl, G., Licciardi, J.M., Murray, A.S., Seidenkrantz, M-S., 2019, Glacial history and palaeo-environmental change of southern Taimyr Peninsula, Arctic Russia, during the Middle and Late Pleistocene. Earth-Science Reviews 193 (2019), doi.org/10.1016/j.earscirev.2019.04.004.

**Table:** 

**Value of the data** • The comprehensive set of photographs of sediments and their structures provides a reference for interpretation of depositional settings/environments across the Arctic. • The multi-disciplinary approach, combining a large chronometric database from radiocarbon, OSL, ESR, and terrestrial cosmogenic nuclide dating with “classical” palaeontological analyses of flora and fauna sets an example for deciphering the complex succession of glaciations and ice free periods. • Presented data can be used to constrain palaeo-glaciological modelling of the Kara Sea Ice Sheet as part of the Eurasian Ice Sheet for described temporal phases. • The study adds new evidence to ongoing studies of the decisive roles both of this ocean and of the Arctic from a global change perspective.

## 1 Data

**Fig. 1:**
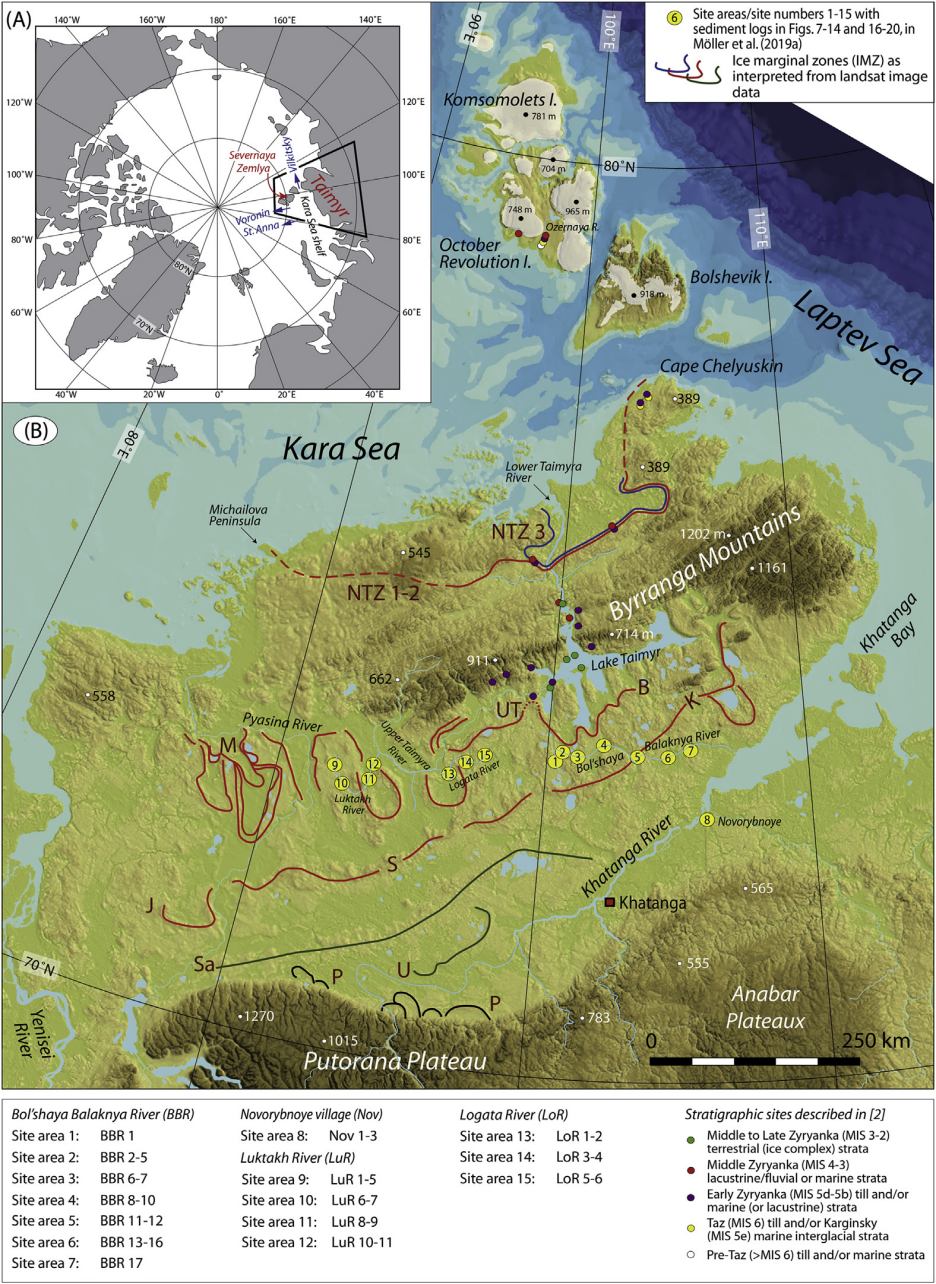
(A) Location map of the Taimyr Peninsula and the Severnaya Zemlya islands. The St. Anna, Voronin and Vilkitsky troughs at the Kara Sea shelf break are marked by blue arrows. (B) Ice-marginal complexes (zones; IMZ) on the Taimyr Peninsula, named according to Kind and Leonov [3], but drawn from Landsat image interpretation by Möller et al. [4] : U = Urdakh, Sa = Sampesa, K = Severokokorsky, J = Jangoda, S = Syntabul, M = Mokoritto, UT = Upper Taimyra and B = Baikuronyora ice marginal zones (IMZ). NTZ = North Taimyr ice marginal zone according to Alexanderson et al. [5]. Lines marked P south and west of the Urdakh IMZ are piedmont glacier moraines, deposited by ice from the Putorana Plateau. Yellow circles, numbered 1–15, mark the position of sites/site areas described stratigraphically in [1] and below in this paper. Small circles color-coded in green, red, purple, yellow and white (chronostratigraphic division) mark positions of stratigraphic sites described in [2]. The base map is from the International Bathymetric Chart of the Arctic Ocean (IBCAO) [6].

**Fig. 2:**
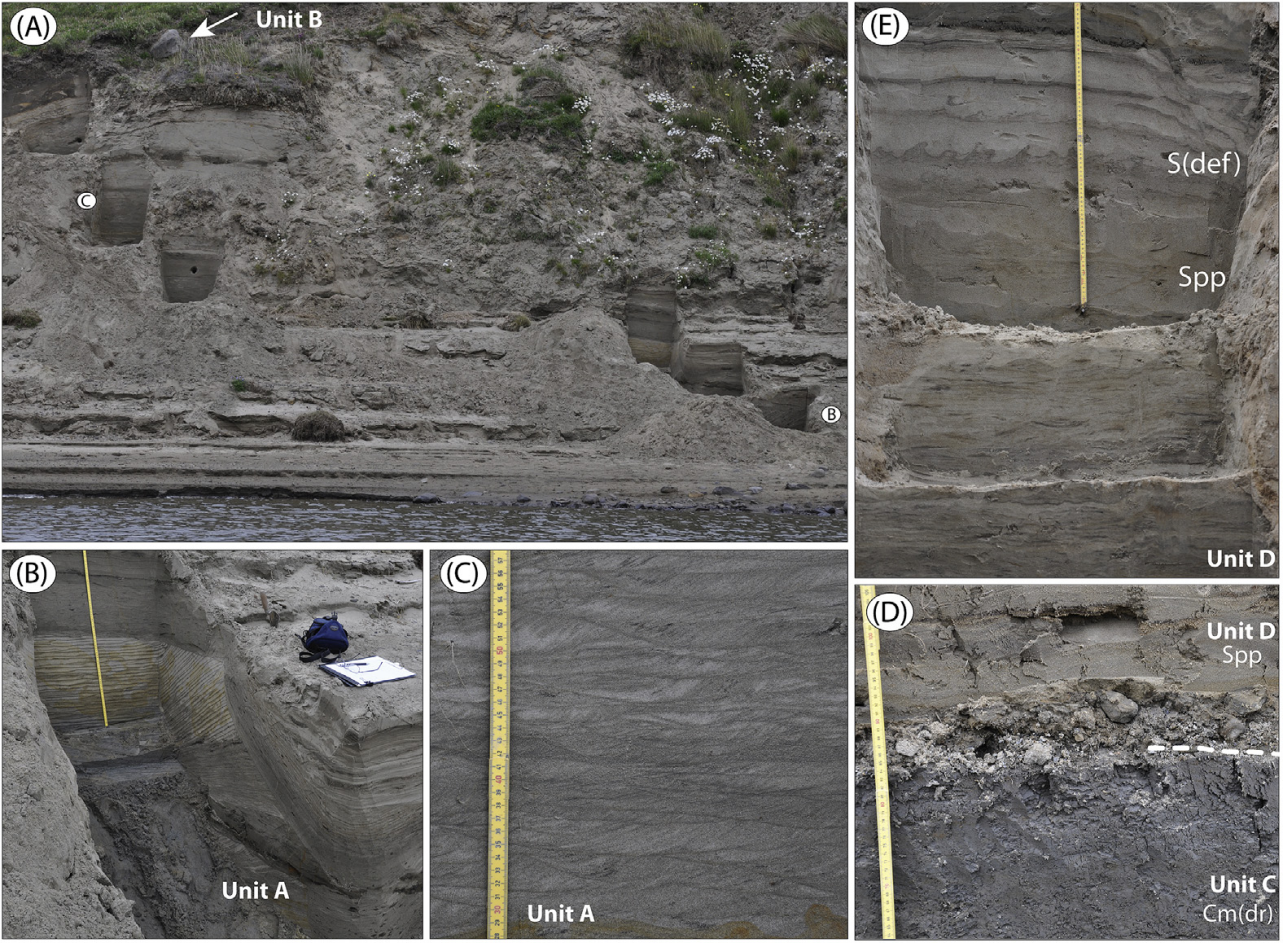
Sediments at site BBR 13 (Fig. 1; sediment log is Fig. 7 in [1]). (A) Overview over the lower part of the section (fluvial sediment unit A). A slumped diamict (unit B) is visible in the upper part. Note large ∼1 m boulder (arrow). (B) At 13–14 m; large-scale trough cross-laminated sand beds (Stc) interbedded with ripple-laminated bedsets (Sr(A)). (C) At ∼ 17 m; small-scale trough cross-lamination in ripple bedsets (Sr(A)). Note organic debris in ripple sets. (D) At ∼33.8 m; contact between glaciomarine unit C clay and shallow marine unit D sand. Note pebbles and cobbles in contact. (E) At ∼35.4 m; unit D planar parallel-laminated sand. Note two sets of load casts, S(def), associated with thin silt beds interbedded with the sand.

**Fig. 3:**
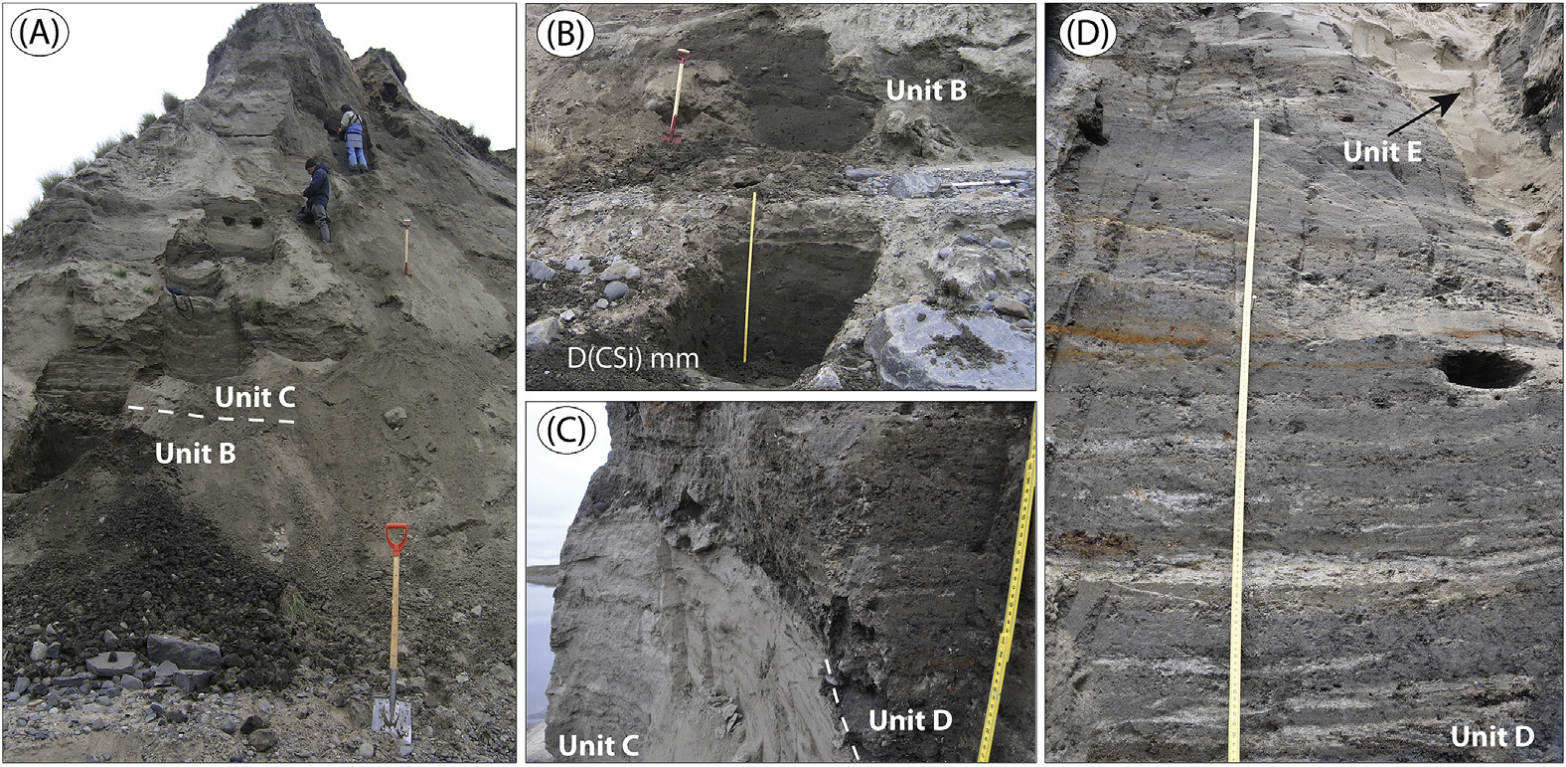
Sediments at site BBR 15 (Fig. 1; sediment log is Fig. 8 in [1]). (A) Overview of the lower part of the section with a diamict (unit B), which is overlain by glaciomarine to shallow marine and C sediments. (B) The unit B diamict. (C) Unit C sand, truncated with a slump erosional surface and overlain with glaciomarine unit D sediment. (D) At ∼22–23 m; interbedded sand and silt in which are frequently occurring ice-rafted clasts (IRD). Note the sand wedge (unit E) that is aeolian sediment infill into a polygonal frost wedge.

**Fig. 4:**
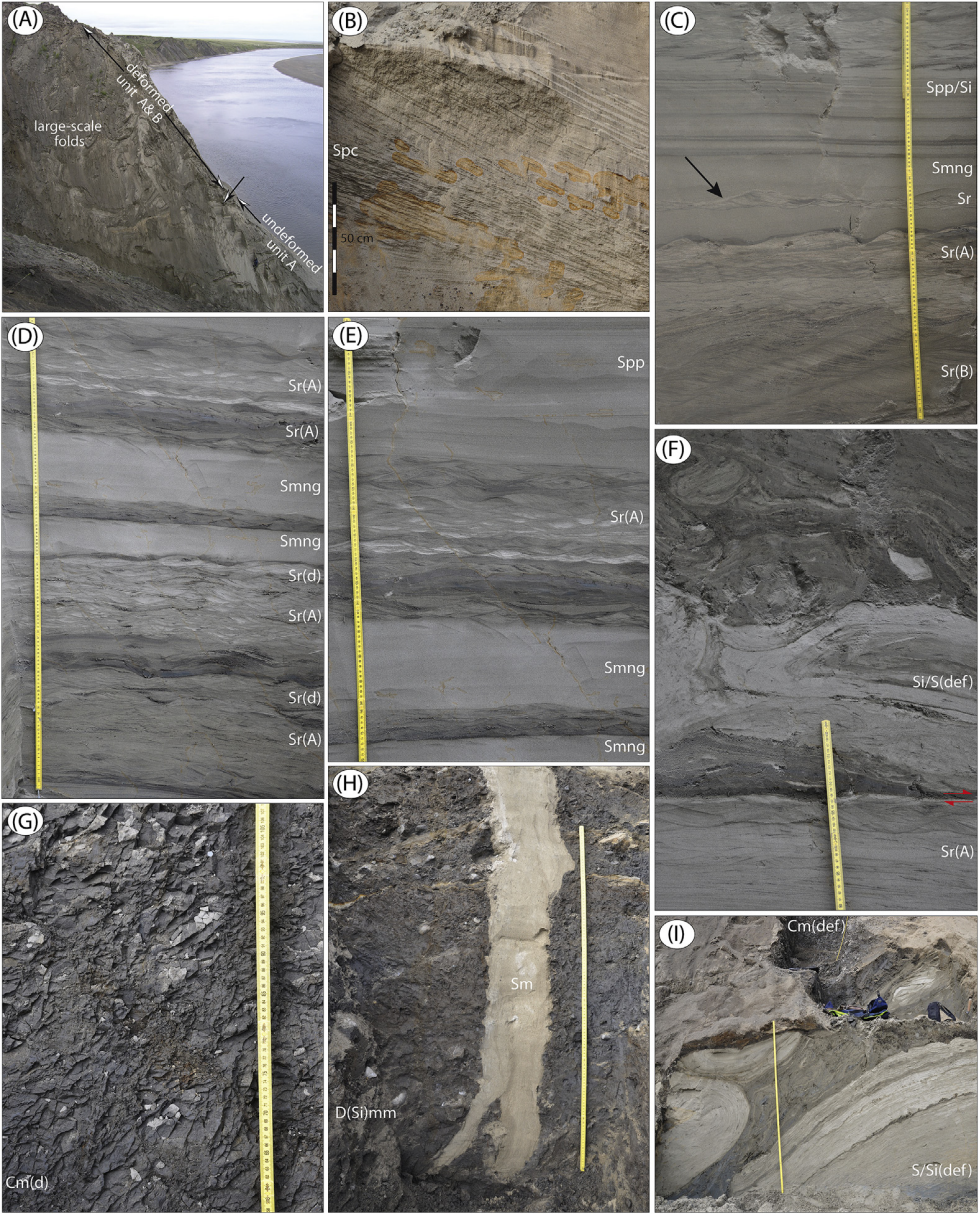
Site Bol'shaya Balaknya 16. (A) The 35 m high river-cut cliff at BBR 16 (Fig. 1; sediment log is Fig. 9 in [1]). Undeformed unit A fluvial sediments are indicated, over which is ∼15 m of glaciotectonically deformed fluvial and marine sediment. (B) Bar cross-laminated sand (unit A), deposited in a shallow marine setting. (C) Climbing type-B ripple lamination, Sr(B), with silt draping, on top of which is sand with planar parallel-lamination and massive, normally graded sand (unit A), deposited in a shallow marine setting. The arrow indicates an interbedded ripple form set. (D–E) Stacked successions of interbedded ripple-laminated sand, Sr(A), often with draping silt, and massive, normally graded sand beds (unit A), deposited in a shallow marine setting. (F) Undeformed ripple-laminated sand (unit A), which above a decollement surface (red arrows) are strongly deformed with a stress transfer from SE. (G) Marine clay (unit B). (H). At ∼38–39 m; unit C diamict with a prominent sand wedge (unit D), that is aeolian sediment infill into a polygonal frost wedge. (I) Large-scale tectonics into unit A sediment (∼31 m).

**Fig. 5:**
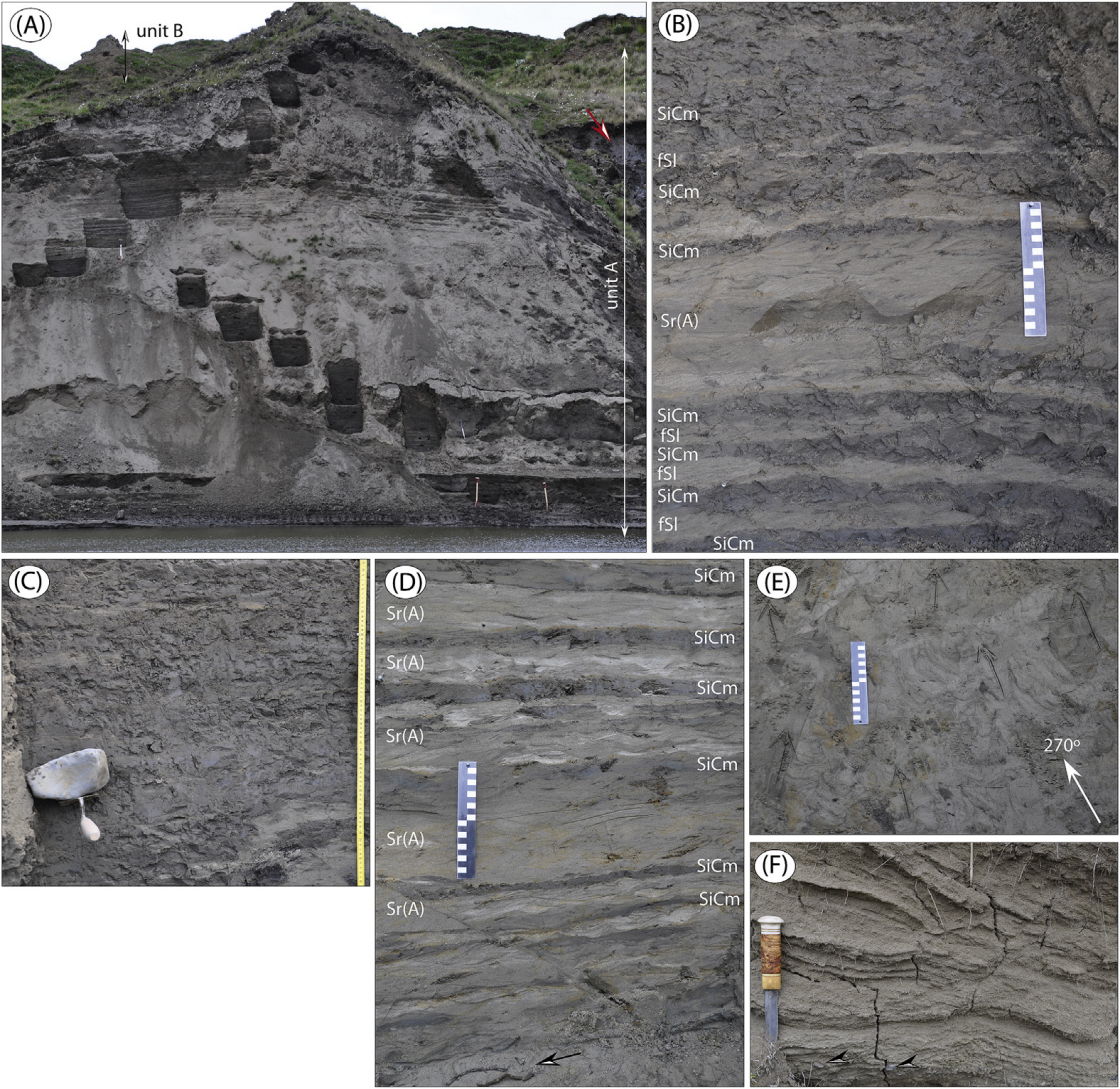
Sediment succession at site BBR6 (Fig. 1; sediment log is Fig. 13 in [1]), exposing marine sediments (unit A) below fluvial sand (unit B). Note the pillar-like topography of the upper part of the cliff that is due to ravine formation along melting ground-ice wedges, emanating from unit B (ground ice at red arrow). (B) Massive silty clay interbedded with thin fine sand beds and a thicker set of ripple-laminated sand (∼37 m). (C) Massive silty clay with drop-stone (IRD) of 14*9 cm (∼37.7 m). (D) Stacked sequence of ripple through cross-laminated sand, interbedded with thinner beds of massive silty clay (∼45 m). Note organic debris both in clay beds and ripple troughs. Some of this material includes twigs with diameters of 3–5 mm (arrow point to such twigs excavated, lying on the trench bottom). (E) Horizontal surface in dug sediment pits, showing the trend and thus palaeo-flow direction of ripple troughs (drawn arrows; mean direction towards 270°). (F) Unit B planar parallel-laminated sand with out-sized pebbles (two indicated by arrows). Sediment slumped at digging and thus most clearly displays internal structures in wind-weathered, coherent surfaces before excavation.

**Fig. 6:**
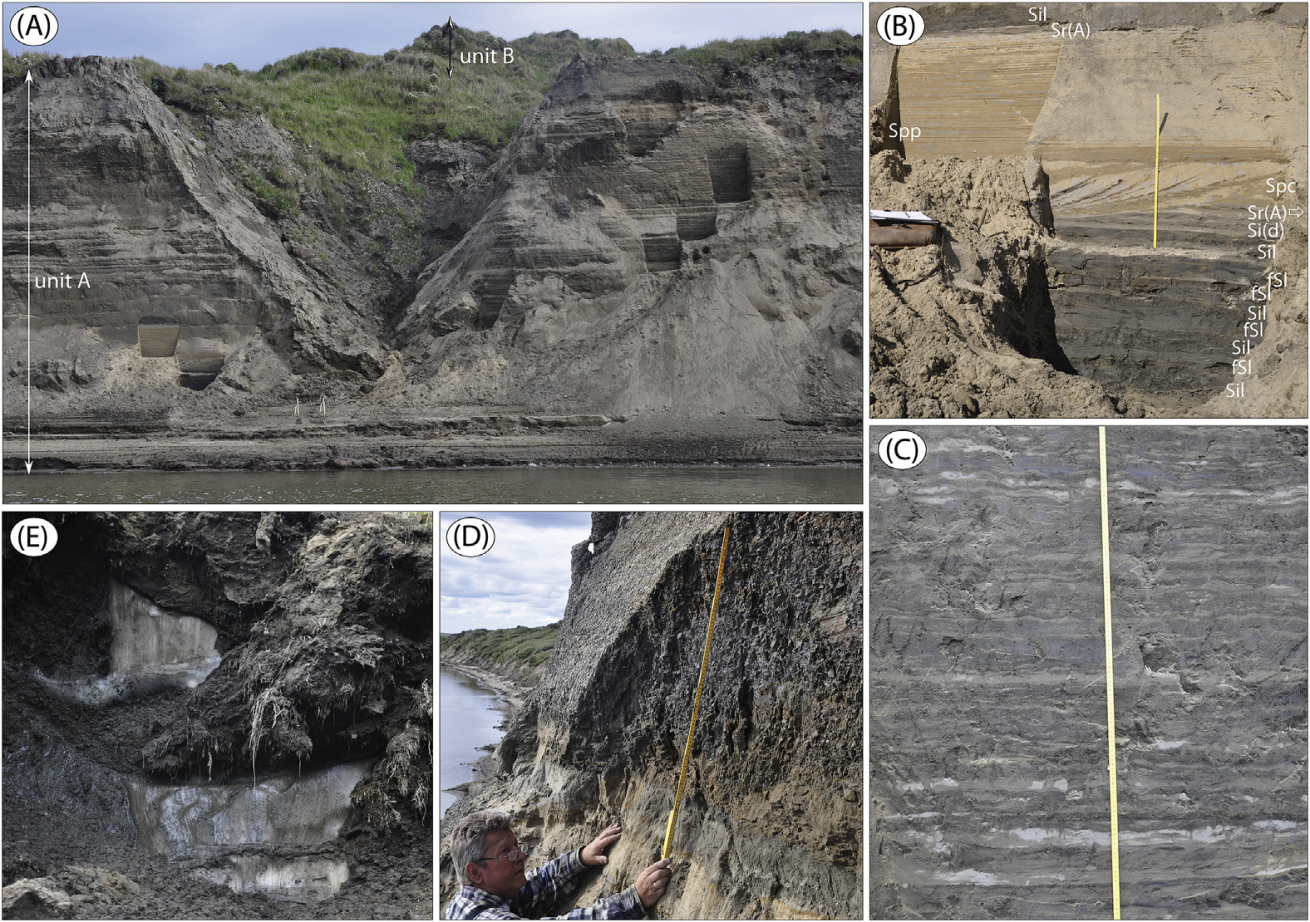
(A) Sediment succession at site BBR 8 (Fig. 1; sediment log is Fig. 14 in [1]) exposing marine sediments (unit A) below terrestrial ice complex deposits (unit B). Note the pillar-like topography of the upper part of the cliff (baydjarakhs) that is due to melting of ground-ice wedges. (B) Lower part of unit A with interbedded laminated silt and fine sand, cross laminated sand with organic debris layers and overlain by a thick bed of planar parallel-laminated sand (∼37.6–40 m). (C) Interbedded laminated silt and thin sand beds, some of them as ripple form sets (starved ripples) (∼45–46 m). (D) Contact (∼48.7 m) between massive sand (unit A1) and laminated clay (unit A2). (E) Silty peat with intraformational ground-ice wedges (ice complex), unit B.

**Fig. 7:**
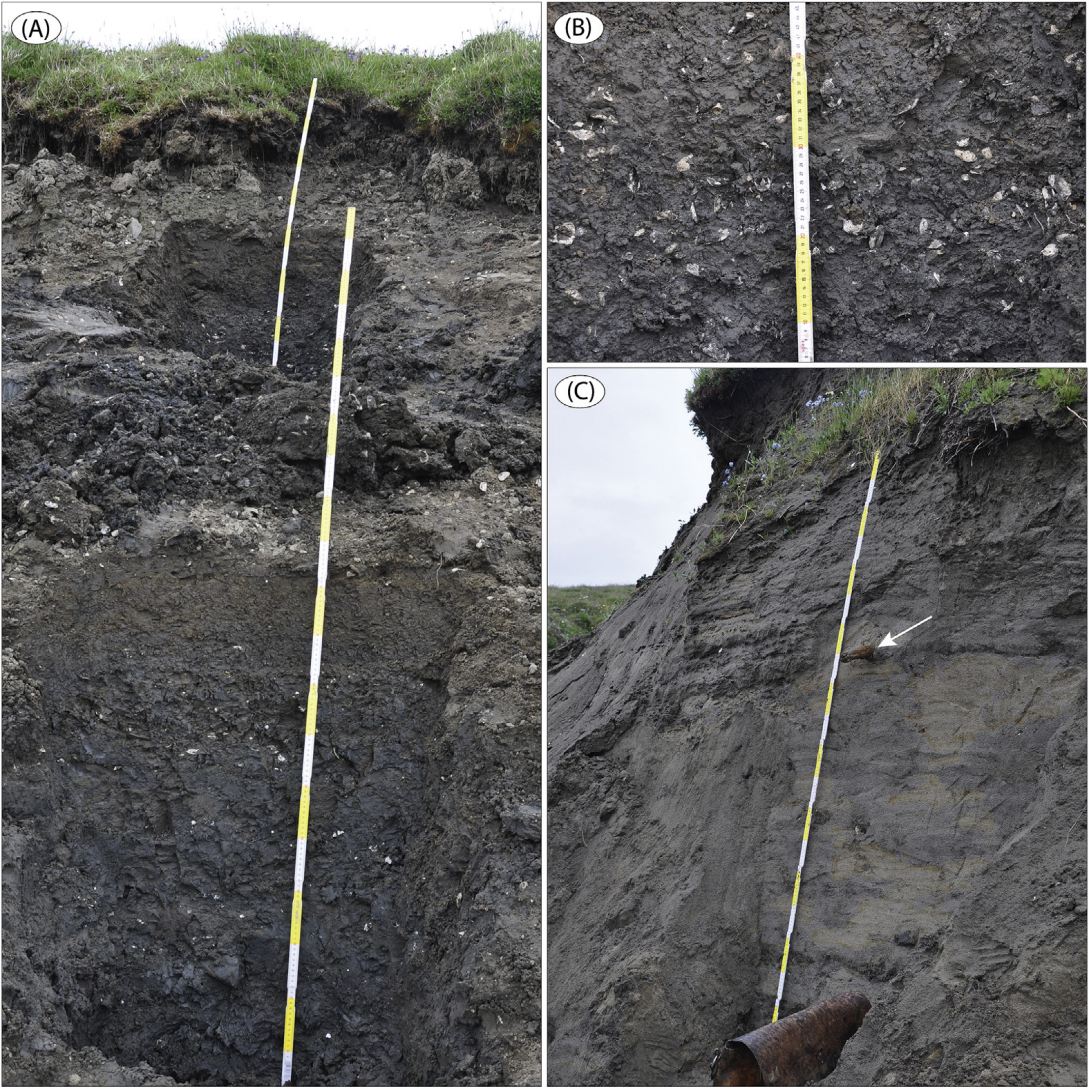
(A) Massive silty clay at site LuR 3 (Fig. 1; sediment log is Fig. 16 in [1]), rich in out-sized drop stones (IRD) and with an abundance of *in situ* molluscs. (B) Horizon with very high abundance of both paired *in situ* and redeposited (single shells) molluscs (∼59.1 m, LuR 3). (C) Planar parallel-laminated fine sand in the upper part of section LuR 4 (Fig. 1; sediment log is Fig. 16 in [1]). The sand is rich with in situ-positioned molluscs. Note the embedded wood twig (diameter ∼5 cm) at white arrow.

**Fig. 8:**
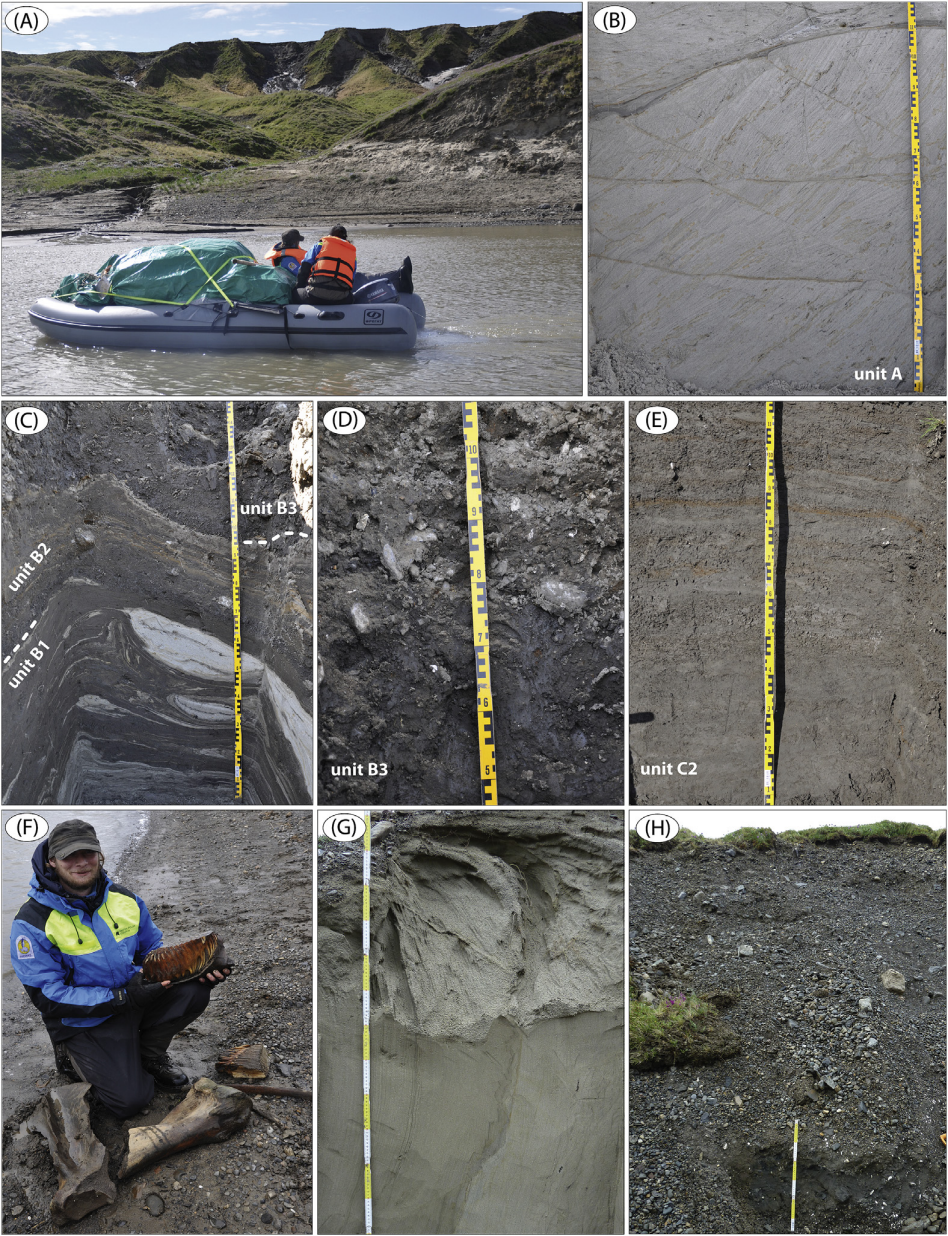
(A) Solifluction ravines at site LuR 6 (Fig. 1; sediment log is Fig. 17 in [1]). Sediment thickness above river is ∼30 m. (B) LuR 6a, unit A: planar laminated sand, glaciotectonically imbricated and thrust from northeast. (C) LuR 6a, unit B1 glaciotectonite: deformed silt with folded inclusion bodies (boudinage). (D) LuR 6a, unit B2: massive silty clayey diamict (traction till). (E) LuR 6a, unit C2: faintly laminated glacio-marine silt. (F) Mammoth remains eroded at Luktakh river side (site LuR 7) out of soliflucted ‘ice complex’ sediment. (G) Unit A sand at LuR 9a (Fig. 1; sediment log is Fig. 17 in [1]). Vertically standing sand displays at its top an overturned fold with vergence towards SSW (logs in Fig. 17, in [1]). (H) Unit B marine sand and cobble gravel beds at LuR 9b (Fig. 17 in [1]). Note the high abundance of mollusc shells visible at the base of the section.

**Fig. 9:**
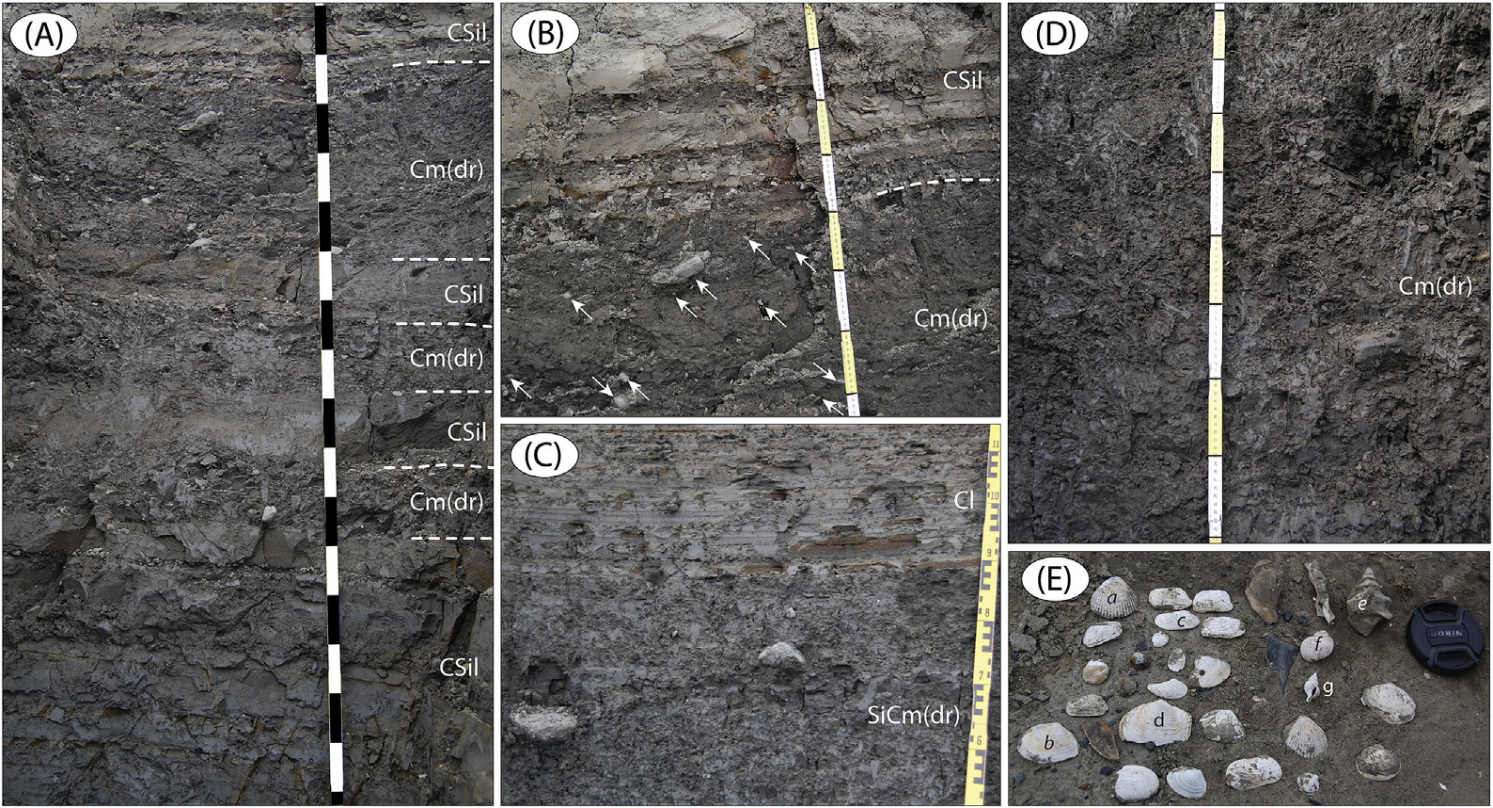
Sediments exposed at site LoR 2 (Fig. 1; sediment log is Fig. 18A in Fig. 17 in [1]). (A) Interbedded massive clay with drop stones (IRD) and laminated clayey silt (31–33 m; ruler in 10 cm intervals) (B) Enlargement of upper the part of (A), 32.5–33.0 m, a few of the frequent drop stones (IRD) marked by white arrows. (C) Massive silty clay with drop stones (IRD), with laminated clay on top (27.1–27.8 m). (D) Massive silty clay with drop stones (IRD) (22.0–22.8 m). (E) Molluscs encountered in the marine sediments of LoR 2. Frequent bivalves are *Ciliatocardium ciliatum* (a), *Macoma calcarea* (b), *Hiatella arctica* (c) and *Mya truncata* (d). Gastropodes include *Neptunea despecta* (e), *Amauropsis islandica* (f) and *Trophon clathratus* (g).

**Fig. 10:**
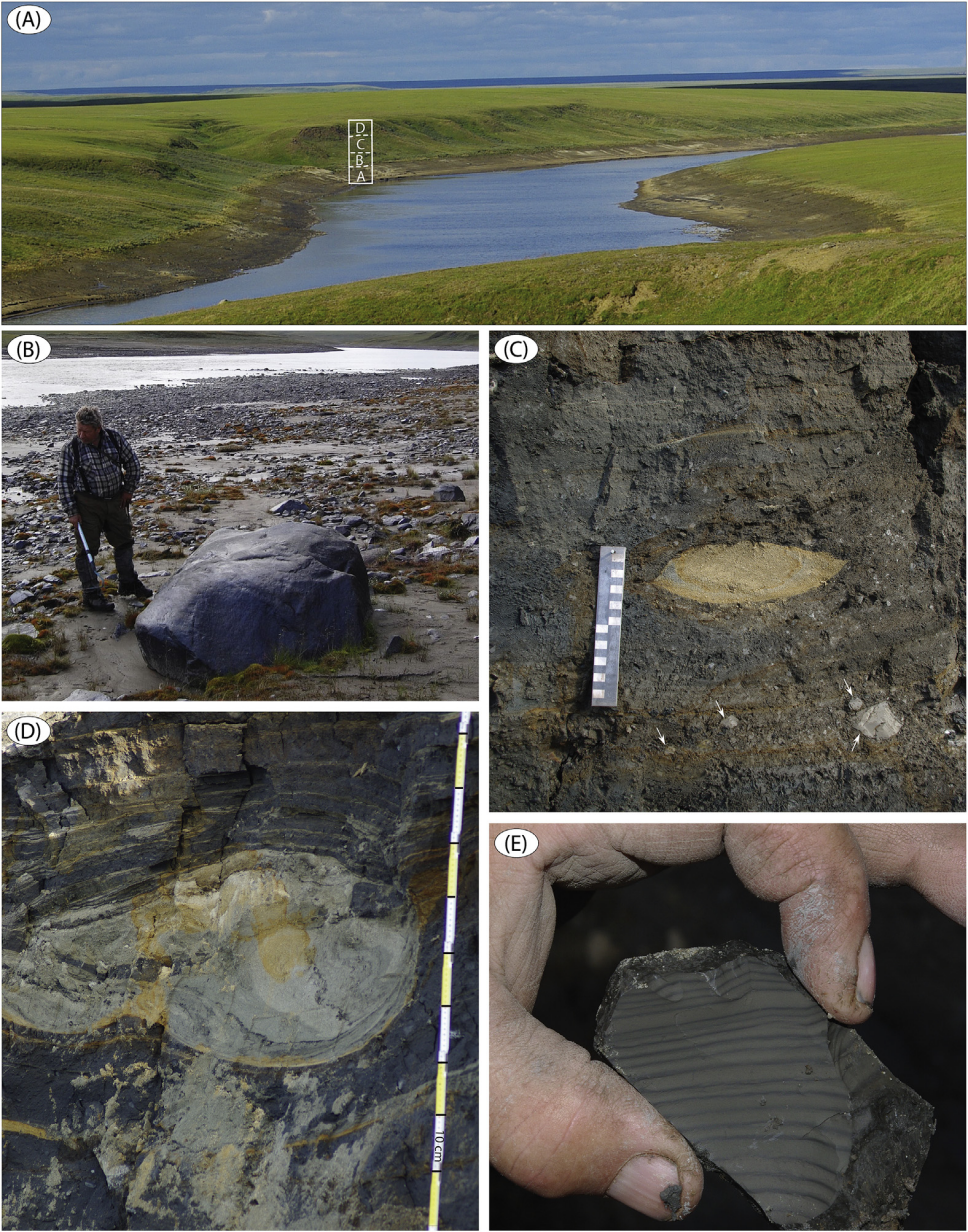
(A) North bank of the Logata River at site LoR 5 (Fig. 1; sediment log is Fig. 15B in Fig. 17 in [1]). Four sediment units (A–D) were identified from shallow test pits in the ∼15 m high slope above the river. (B) Boulder and cobble armour of the river beach below the high-water mark at site LoR 5; the clasts result from erosion into the unit B diamict. (C) Close-up of the glacio-tectonically laminated diamict (unit B) at site LoR 6 (Fig. 1; sediment log is Fig. 18 in Fig. 17 in [1]). Note lenticular sand intraclast (boudin) and the more angular, finely intra-laminated clay intraclasts (marked by small white arrows). (D) Sand intraclast (boudin) with internal primary lamination conforming to its outer shape; unit B diamict at site LoR 6. (E) Close-up of one of the clay intraclasts with preserved intra-lamination (2–5 mm) found in the unit B diamict at site LoR 6.

**Fig. 11:**
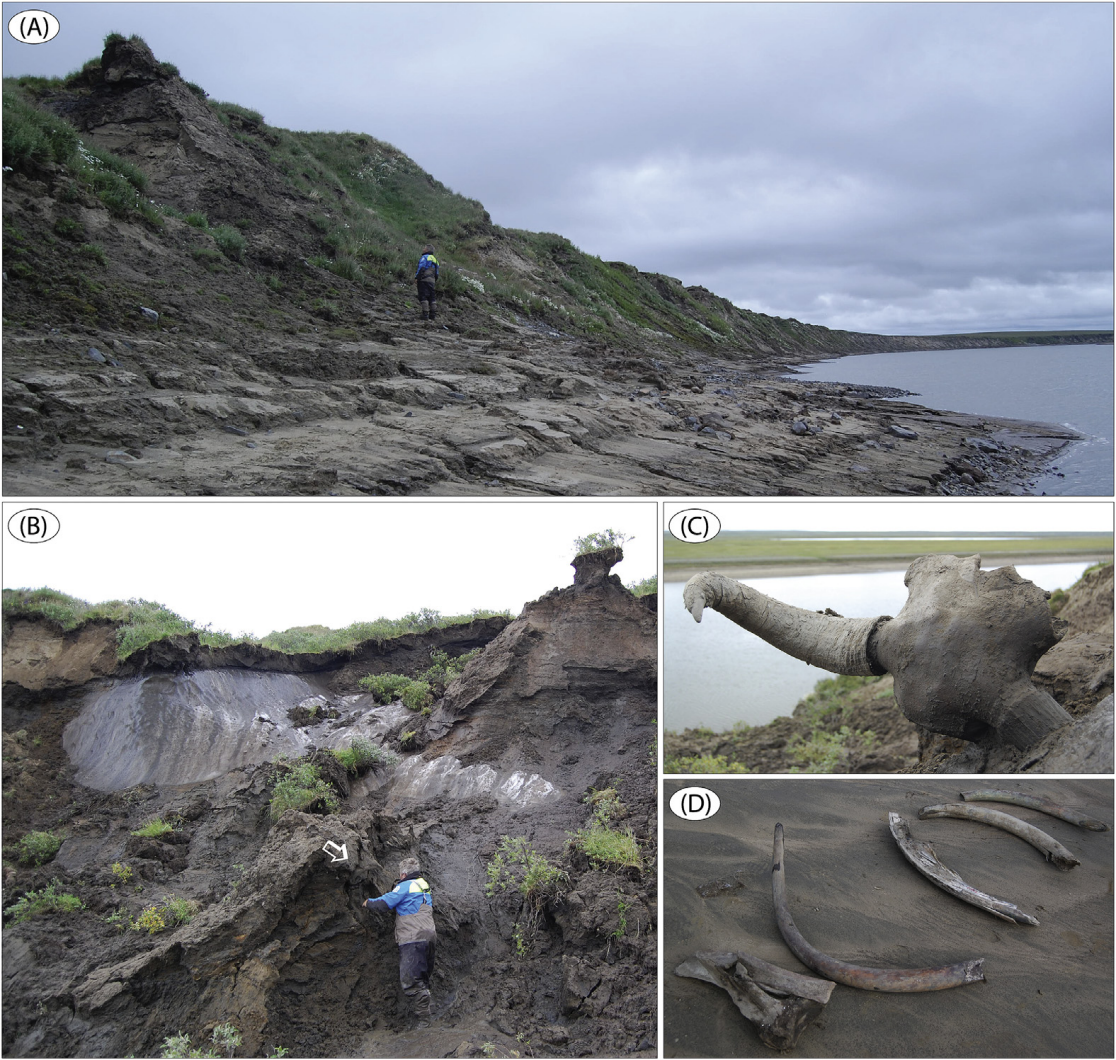

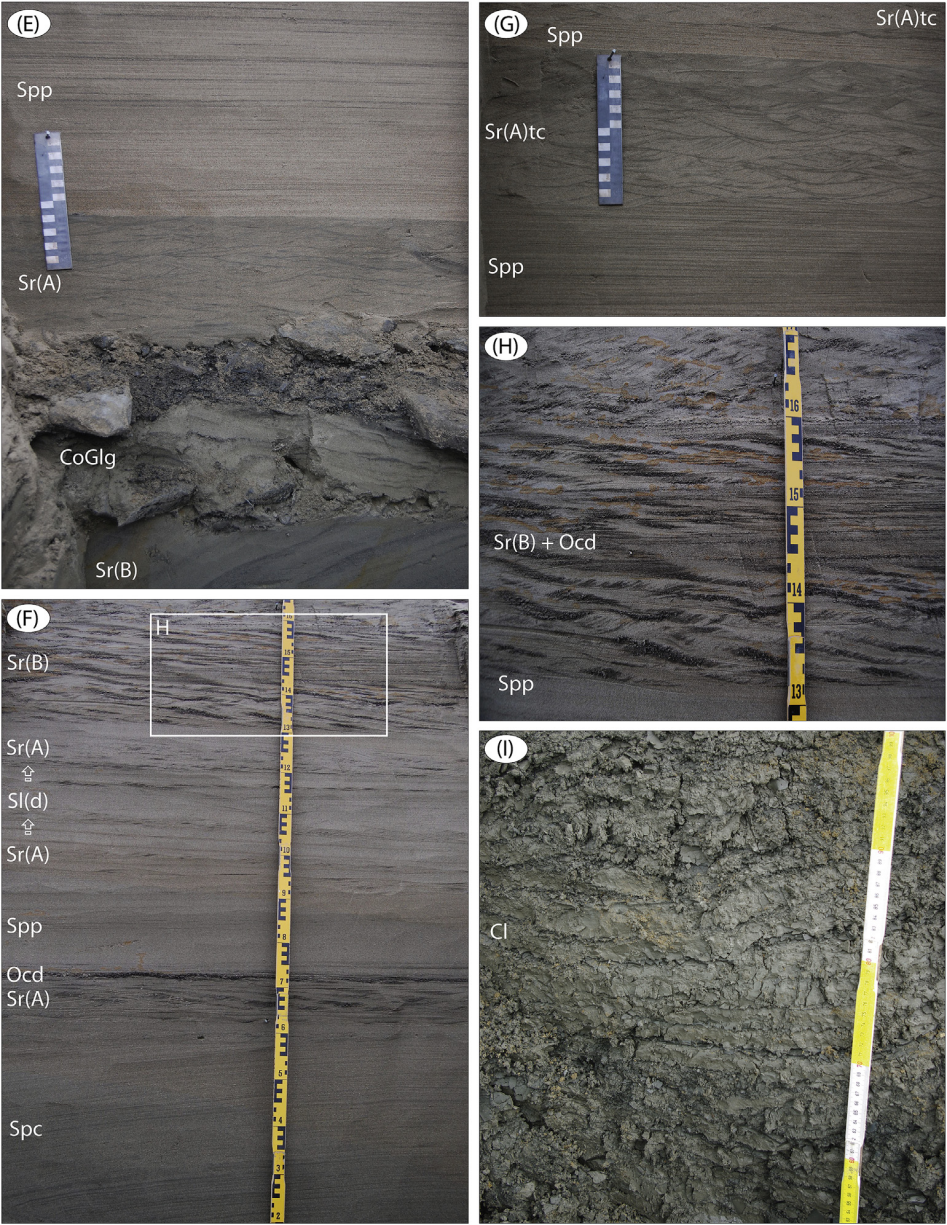
Site Logata River 3 (Fig. 1; sediment logs are in Fig. 19 in [1]). (A) The 2 km long river cliff with sediments documented at four sites LoR 3a-d. (B) Topmost unit D (LoR 3d) which is ‘ice-complex’ silt, rich in organic debris and with syngenetic ice wedges. An arrow indicates the skull of step bison (C) together with a high number of other bison skeleton parts, suggesting that a mostly intact animal body is present in the sediments. (C) Partly melted-out step bison (*Bison priscus*) skull; age is c. 43 cal ka BP. (D) Megafauna remains (mammoth tusks and scapulas), sampled on the river beach below outcropping ice-complex sediment at site LoR 3. (E) LoR 3a, ∼32–33 m (unit D); syndepositionally block-slumped ripple laminated sand, with post-slump erosion (CoGlg), followed by alternating Spp and Sr(A) beds. (F) LoR 3, ∼27.4–28.8 m (unit D); interbedded planar cross-bedded, planar parallel-laminated and ripple laminated sand. Note the high content of organic debris in some beds, seen up-scaled in panel H. (G) LoR 3a, ∼33–34.4 m (unit D); planar parallel-laminated sand interbedded with ripple trough cross-laminated sand. (H) Up-scaled upper part of (F) with Sr(B) sand with a high organic debris content in ripple troughs and foresets. (I) LoR 3b, ∼24.5–25 m (unit C); marine, rhythmically laminated clay.

**Fig. 12:**
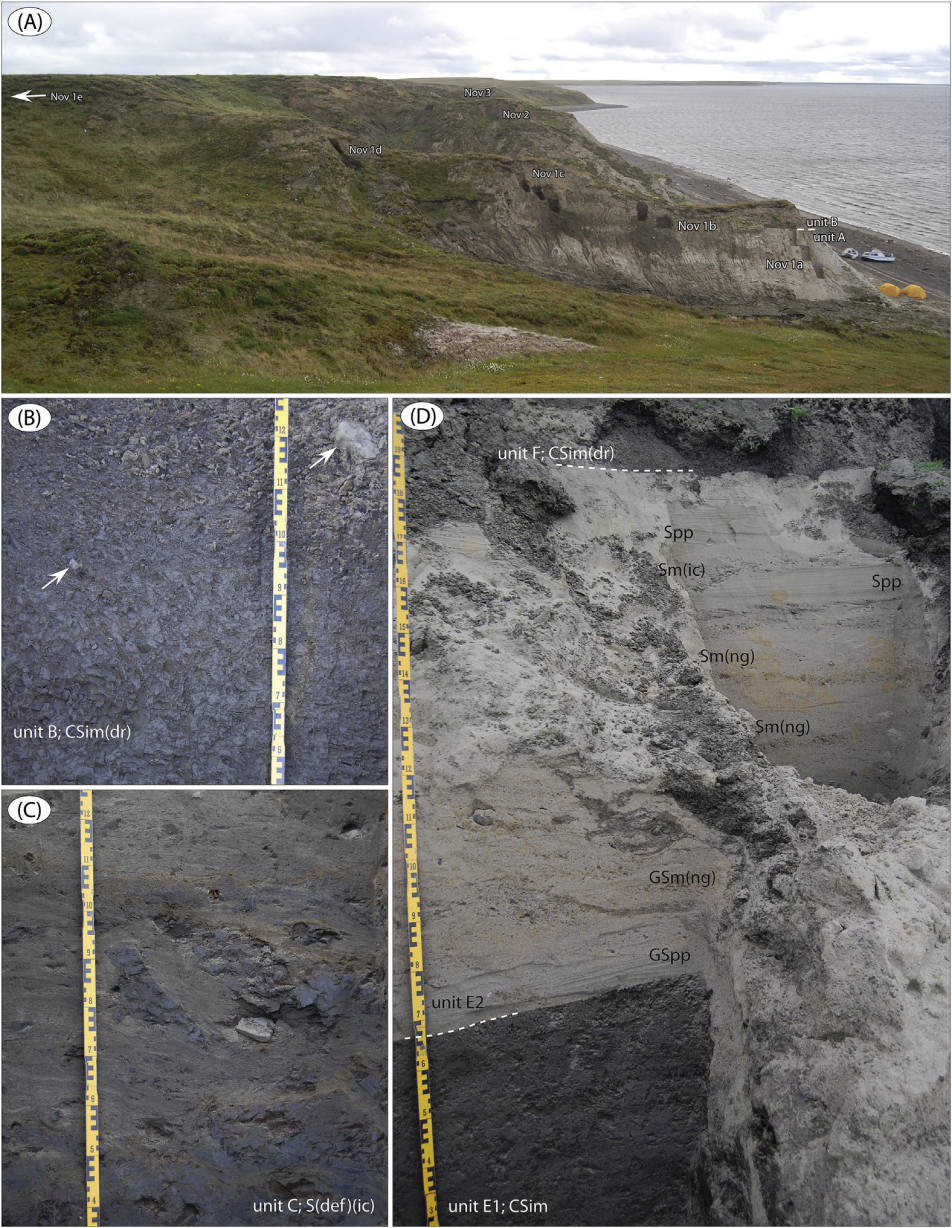
The Novorybnoye site (Fig. 1; sediment logs are in Fig. 20 in Fig. 17 in [1]). (A) Overview of the river cliff at Novorybnoye (looking east). The boundary between the Cretaceous sand (unit A) and overlying Quaternary sediment succession (unit B) is market by hatched line, as well as position of logged sub-sections (Nov 1a-e) and main sections (Nov 2 and Nov 3). (B) Glaciomarine unit B (Nov 1b, ∼13.5 m); massive, mollusc-bearing clayey silt with ice-rafted drop stones. (C) Unit C (Nov 1c, 14–15 m); shear laminated sand with intraclasts (boudins) from the unit B sediments; a glaciotectonite. (D) Unit E and F at site Nov 2; marine clayey silt overlain by shallow marine sand, in turn overlain by glaciomarine clayey silt with ice-rafted drop stones.

The data presented here and in Möller et al. [1] come from studies of sediment exposures along the Bol'shaya Balaknya and the Luktakh – Upper Taimyra – Logata river systems on the southern part of the Taimyr Peninsula, NW Siberia (Fig. 1), and from a complex of sites situated on the southern shore of the Khatanga River close to the small settlement of Novorybnoye (site 8, Fig. 1). Fig. 2, Fig. 3, Fig. 4, Fig. 5, Fig. 6, Fig. 7, Fig. 8, Fig. 9, Fig. 10, Fig. 11 illustrate the general morphology and typical examples of sediments found at our sites. Table 2, Table 3, Table 4 contain results of analysis of foraminifera, mollusc faunas and plant and animal remains. Table 5, Table 6, Table 7 contain chronological data (radiocarbon ages, Electron Spin Resonance (ESR) ages, Optically Stimulated Luminescence (OSL) ages) on logged sedimentary units, and Table 8, Table 9, Table 10 contain data on terrestrial cosmogenic nuclide (TNC) ^36^Cl exposure ages on erratic boulders sampled from the top of mapped Ice Marginal Zones (IMZs) (see Fig. 12).

## 2 Experimental design, materials and methods

### 2.1 Sedimentology and stratigraphy

We focused on laterally extensive river bluff sections for sedimentological and lithostratigraphical descriptions, and targeted geochronological sampling. The sections were dug out in a stair-case manner (see Fig. 5B in [1]) in which sediment composition and structures were logged mostly at 1:10 scale (all site logs are in [1]). A number of images are presented below as examples of sediment composition and structures, and references to these are given in the site descriptions in [1]. Lithofacies codes in photographs are according to Table 1.

### 2.2 Foraminiferal analyses

Selected sites with marine or possibly marine strata were sampled for foraminiferal analyses. A total of 129 samples from eight sections (sections BBR 6, 8, 12, 13, 15, 16, 17, Nov 1 and LuR 6; Fig. 1) were collected. The samples were processed at the Dept. of Geoscience, Aarhus University, Denmark, using 40–160 g of dry sediment (most commonly 90–140 g). The samples were wet-sieved using tap-water and sieve sizes with mesh diameters of 63, 100 and 1000 μm, cf. [8], and dried in an oven at 40 °C. The foraminifera in the 100–1000 μm fraction were subsequently concentrated using the heavy liquid C_2_Cl_4_ (density of 1.6 g/cm^3^), collected and taxonomically identified. Unfortunately, most samples proved barren; only very few foraminiferal specimens were found in only two of the sections and only benthic foraminifera were present (Table 2).

**Table 1:** Lithofacies codes (1st, 2nd and 3rd order code system) and their description as used in this work (basic system according to Eyles et al. [7]).

Private Lithofacies code:	Lithofacies type description: Grain size, grain support system, internal structures
*Diamictons:*	
D(G/S/Si/C)	Diamicton, gravelly, sandy, silty or clayey. One or more grain-size code letters within brackets
D( )mm	Diamicton, matrix-supported, massive
D( )ms	Diamicton, matrix-supported, stratified
D( )mm/ms(s)	Diamicton, …., sheared
D( )ms(a)	Diamicton, …., attenuated
D( )mm(ng)	Diamicton, matrix-supported, massive, normally graded
D( )mm(ig)	Diamicton, matrix-supported, massive, inversely graded
*Sorted sediment facies, 1*st *code on grain size:*	
B, Co, G, CoG, G, SG, GS, S, Si, C	Boulder, Cobble, Cobble-gravel, Gravel, Gravelly-sandy, Sand, Silt, Clay facies
*Sorted sediment facies, 2*nd *code on clast support system and internal lamination:*	
-- cm	clast-supported, massive
-- mm	matrix-supported, massive
-- m	massive
-- pp	planar parallel-laminated
-- l	laminated (silt, clay)
-- dp	delta planar-laminated
-- tc	trough cross-laminated
-- pc	Planar cross-laminated
-- r	Ripple
-- r(A), r(B)	type A, type B ripple laminated
-- r(d)	draped ripple lamination
-- lg	stringer, lag, erosion remnant
*Sorted sediment facies, 3*^*d*^*code:*	
(o)	organic-rich
(ic)	intra-clasts (e.g., silt, clay in sand)
(bi)	bimodal composition
(im)	imbricated clast axes
(ng), (ig)	normally graded, inversely graded
(b)	burrows, bioturbated
(def)	deformed
(dr)	drop clasts (IRD)
*Organic sediment, 1*st *code:*	
O	Organic matter, unspecified
P	Peat
*2*nd *code:*	
cd	coarse detritus
fd	fine detritus

**Table 2:** Foraminiferal counts provided as raw count data in the actual sample. Only samples from the parts of the sections, where foraminifera are present, are included. Author names of taxa are also given. Of seven sections along the Bol'shaya Balaknya River, sampled for foraminiferal analyses (sections BBR 6, 8, 12, 13, 15, 16, 17), and the Novorybnoye 1 section (Fig. 1), all but two were found barren. Section LuR 6 along the Luktakh River (Fig. 1) was only analysed for foraminifera in it lowermost unit A, but not in marine sediments further up (unit C) in the sediment succession. Section logs are found in Figs. 7, 8, 9, 12, 13, 14 and 17 in Möller et al. [1].

Site	BBR 6 (Fig. 13)											BBR 15 (Fig. 8)		LuR 6 (Fig. 17)
*Sample height (m a.s.l.)* Sediment unit Sample size (gram dry sediment)	*38.5* A1 114	*39.0* A1 105	*39.5* A1 118	*40.0* A1 134	*40.5* A1 115	*41.0* A1 125	*41.5* A1 137	*42.0* A1 127	*42.5* A1 141	*43.0* A1 122	*43.5* A1 123	*21.0* D 128	*21.5* D 146	*24.3* A c. 1200
**Benthic foraminiferal taxa**														
*Astrononion gallowayi* Loeblich & Tappan, 1953	*-*	*-*	*-*	*-*	*-*	*-*	*-*	*-*	*-*	*-*	*-*	*-*	*-*	1
*Buccella frigida* (Cushman, 1922)	1	7	7	*-*	11	*-*	29	4	–	–	–	2	–	1
*Cassidulina reniforme* Nørvang, 1945	–	–	–	–	–	–	–	–	–	–	–	–	–	4
*Cibicides lobatulus* (Walker & Jacob, 1798)	–	–	–	–	–	–	–	–	–	–	–	–	–	2
*Cibicides scaldisiensis* Ten Dam & Reinhold, 1941	–	–	–	–	–	–	–	–	–	–	–	–	–	1
*Elphidium albiumbilicatum* (Weiss, 1954)	6	–	1	1	2	4	–	17	–	–	–	2	–	–
*Elphidium asklundi* Brotzen, 1943		–	–	–	–	–	–	–	–	–	–	–	–	5
*Elphidium bartletti* Cushman, 1933	–	*-*	*-*	*-*	*-*	*-*	–	–	–	–	–	–	–	48
*Elphidium clavatum* Cushman, 1930	–	–	–	–	–	1	–	1	–	–	1	2	4	30
*Elphidium hallandense* Brotzen 1943	*-*	*-*	*-*	*-*	*-*		–	–	–	–	–	–		1
*Elphidium ustulatum* Todd, 1957	–	–	–	–	–	–	–	–	–	–	–	12	16	4
*Elphidiella hannai* (Cushman & Grant, 1927)	*-*	–	–	–	–	–	–	–	–	–	–	–	2	
*Elphidiella groenlandica* (Cushman, 1936)	1	–	6	6	–	–	1	1	–	–	–	–	–	
*Eilohedra vitrea* (Parker, 1953)		*-*	*-*	*-*	*-*	*-*	*-*	*-*	*-*	*-*	*-*	*-*	*-*	1
*Glabratella* sp.		–	–	–	–	–	–	–	–	–	–	2	3	–
*Haynesina orbiculare* (Brady, 1881)	–	1	3	3	5	12	4		–	–	–	7	13	33
*Islandiella helenae* Feyling-Hanssen & Buzas, 1976	*-*	*-*	*-*	*-*	*-*	*-*	*-*	*-*	*-*	*-*	*-*	*-*		2
*Islandiella inflata* (Gudina, 1966)		*-*	*-*	*-*	*-*	*-*	*-*	*-*	*-*	*-*	*-*	*-*	*-*	2
*Stainforthia loeblichi* (Feyling-Hanssen, 1954)	*-*	*-*	*-*	*-*	*-*		–	–	–	–	–	–		1
Polymorphinidae		–	–	–	–	–	–	1	–	–	–	1	2	–
Indeterminated		–	–	–	–	–	–	1	–	–	–	–	–	–
**Planktonic foraminiferal taxa**														
*Neogloboquadrina dutertrei*		*-*	*-*	*-*	*-*	*-*	*-*	*-*	*-*	*-*	*-*	*-*	*-*	1
*Neogloboquadrina pachyderma (sinistral)*	*-*	*-*	*-*	*-*	*-*	*-*	*-*	*-*	*-*	*-*	*-*	*-*	*-*	1
**Other**														
Ostracod valves		–	–	–	–	–	–	–	4	–	–	2	–	–

**Table 3:** Mollusc faunas from sites BBR 6, 8, 13, 14, 15, 17, LuR 1–3, LuR 5, 6 and LoR 2. Section logs for these sites are found in Figs. 7, 8, 11, 13, 14, 16, 17 and 18 in Möller et al. [1].

Species:	Bio-geography class	BBR 6:0; 39–51 m	BBR 8:5; 43–44 m	BBR 8:8; 46–47 m	BBR 13; 31–33,5 m	BBR 14:0; 28–30 m	BBR 15A:0; beach sample	BBR 15A:2; 21–23 m	BBR 15A:4; 20.1 ± 0.1 m	BBR 17; 8–12 m	LuR 1:1; 51–53 m	LuR 2:2; 52–54 m	LuR 3:3; 59 ± 0.5 m	LuR 5:3; 58–59 m	Lu R 6a:2; 48–49 m	Lu R 6a:3; 46–47 m	LuR 6a:4; 44–45 m	LuR 6b:3; 31–32 m	LuR 6b:4; 30 ± 0.5 m	Logata 2:6
**Gastropods**	N/A																			
*Solariella obscura* (Couthouy, 1838)	N/A															+				
*Tachyrhynchus erosus* (Couthouy, 1838)	N/A														•					
*Euspira pallida* (Broderip & Sowerby, 1829)	N/A	+								••								+		
*Amauropsis islandica* (Gmelin, 1791)	N/A									••										+
*Boreotrophon clathratus* (Linné, 1767)	N/A																+			+
*Buccinum undatum* (Linné, 1758)	*SA*										+						+			
*Oenopota* sp.																+				
*Buccinum* sp.	N/A							+												
*Neptunea despecta* (Linné, 1758)	*A*	+								•					••	••				+
*Admete viridula* (Fabricius, 1780)	N/A																+			
*Retusa obtusa* (Montagu, 1803)	*?*															+				
*Cylichna alba* (Brown, 1827)	*?*															+				
**Bivalves**																				
*Eunucula tenuis* (Montagu, 1808)	N/A																+			+
*Nuculana pernula Müller, 1779*	N/A																			+
*Portlandia arctica* (Gray, 1824)	*A*	+								•••										
*Mytilus edulis* (Linné, 1758)	*SA*									•					•					
*Musculus* sp.																+				
*Similpecten greenlandicum* (Sowerby, 1842)	*A*													•						
*Chlamys islandica* (Müller, 1776)	*SA*												*+*	••						
*Astarte borealis (Schumacher, 1817)*	*A*	•••			••					••	•	•••		+	+		+			+
*Astarte crenata* (Gray, 1824)	*A*																			+
*Astarte elliptica* (Brown, 1827)	N/A				+															
*Astarte montagui* (Dillwyn, 1817)	N/A						••	••	+											
*Ciliatocardium ciliatum* (Fabricius, 1780)	N/A				•							+	•••	••		••		•		••
*Serripes groenlandicus (Bruguière, 1789)*	N/A															•		+		
*Macoma balthica (Linné, 1758)*	*SA*	•••	•••	•••		•••	+	+		•										
*Macoma calcarea* (Gmelin, 1791)	*A*	+												•	•••	•••	•••	•••		••
*Mya truncata* (Linné, 1758)	N/A										+	••		••		+			••	+
*Hiatella arctica* (Linné, 1767)	N/A	•					•••		••		•••	•••	••	•••	+	+	+	+	•••	•
Cyrtodaria angusta (Nyst & Westendorph, 1839)	*EXT*				+				?											
**Barnacles**																				
*Balanus balanus* (Linné, 1758)	N/A																			
*Balanus crenatus* (Bruguière, 1789)	*A*								+				•			+				
Balanus hameri (Ascanius, 1767)	*SA*								+				+	•	+		+	+		
*Semibalanus balanoides* (Linné, 1758)	*SA*								?						+	+				
Balanoidea								+	+				+	••	+		•			
**Polychaetes**																				
*Polydora ciliata* (Johnston, 1865)	*SA*	+		+								••		•	+		+			+
*Spirorbis spirorbis* (Linné, 1758)	*SA*												+		•	•	+			
**Bryozoans**													+		•	••				
**Algae**																				
*Lithothamnion* sp	N/A													•	+					

No. of valves/fragments: …: >20; ••: 10–19; •: 4–9; +: 1–3; ? dubious identification.

Biogeography classes; SA: subarctic, not present in the area today (grey shaded), A; present in several biogeographic zones, but only dominating in the Arctic. EXT: Extinct.

N/A: widespread in several zones, present in the area today.

**Table 4:** Plants and animals remains from fluvial sediments at site Logata River 3 (LoR 3b and 3d), sediment unit D. Section logs for sites LoR 3 are found in Fig. 19 in Möller et al. [1].

Site/sample:	3b:3	3b:2	3d:3	3d:2	3d:1
m a.s.l.	28.0	31.0	31.6	33.7	34.1
PLANTS					
**Terrestrial**					
*Dryas octopetala* s.l. (L.)	45	2	1	7	1
*Salix herbacea* (L.)	7	–	–	1	1
*Salix* cf. *phylicifolia* (L.)	–	–	–	1	–
*Salix* sp.	–	2	–	–	–
*Ranunculus* sp.	4	–	1	2	–
*Polygonum viviparum* (L.)	–	2	–	–	1
*Rumex acetosella* (L.)	–	–	–	–	1
*Cerastium* sp.	1	–	–	–	–
?*Stellaria* sp.	–	–	–	1	–
*Minuartia* sp.	–	–	–	1	3
*Myosotis alpestris* (F·W. Schmidt)	–	–	–	–	1
?*Draba* sp.	–	–	–	–	2
*Papaver* sect. *Scapiflora*	1	–	–	3	2
*Potentilla* sp.	1	–	–	–	1
*Armeria* sp.	1	–	–	–	–
Poaceae indet.	2	–	–	4	–
*Distichium* sp.	1	–	7	1	–
*Ditrichum* sp.	r	–	–	2	–
*Polytrichum* s. l. sp.	1	–	–	–	–
*Cenococcum geophilum* (Fries)	–	6	12	14	–
**Wetland**					
*Carex* sp.	3	–	–	–	5
*Juncus* sp.	–	–	1	–	3
*Drepanocladus* s.l. sp.	c	a	–	a	c
*Calliergon* sp.	1	–	–	–	–
*Scorpidium* sp.	r	–	c	–	–
*Tomentypnum nitens* (Hedw.) (Loeske)	c	c	–	–	–
**ANIMALS (except Coleoptera)**					
*Daphnia* pulex s.l. (Leydig)	–	–	1	3	–
*Chydorus* cf. *sphaericus* (O·F. Müller)	–	–	2	–	–
*Lepidurus* cf. *arcticus* (Pallas)	1	–	–	–	–
Chironomidae indet.	–	–	3	2	1
Rodentia indet.	8	1	–	–	–
**Coleoptera**					
*Carabus loschnikovi* (Fischer v. W)	–	1	–	–	–
*Nothiophilus aquaticus* (L.)	–	1	–	–	–
*Pterostichus brevicornis* (Kirby)	–	2	–	–	1
*Pterostichus ventricosus Esch.*	–	1	–	–	–
*Amara alpina* (Payk.)	–	–	–	–	1
*Amara Cortonotus* sp.	–	1	–	–	–
*Amara* sp.	–	1	–	–	–
*Harpalus* sp	–	1	–	–	–
*Agabus confinis* (Gyllh.)	–	1	–	–	–
*Apion* spp.	–	2	–	1	–
*Sitona lineellus* (Gyllh.)	–	1	–	–	–
*Sitona lepidus* (Gyllh.)	–	1	–	–	–
*Dorytomus/Anthonomus* sp.	–	–	–	1	–

r: rare, c: common, a: abundant.

**Table 5:** Radiocarbon ages (n = 69) from stratigraphic sections at sites along the Bol'shaya Balaknya River and the Luktakh – Upper Taimyra – Logata river system (Fig. 1). More exact site locations are seen on Fig. 6 and Fig. 15 in Möller et al. [1], and stratigraphic positions of samples are indicated in sediment logs in Möller et al. [1], Figs. 8, 10 11, 13, 14, 16, 18 and 19. Sites with sediment units marked with (*) are not described in [1], but will be used in a forthcoming paper. Finite radiocarbon ages on terrestrial material have been recalculated to calibrated^14^C years by software package Oxcal v4.3.2 [12] with use of IntCal 13. LuS datings were conducted at the Radiocarbon Dating Laboratory, Department of Geology, Lund University, Sweden, while the ЛУ –labelled datings (BBR 8) were conducted at the Geomorphology and paleogeography of Polar regions and Wold Ocean Laboratory, St. Petersburg State University, Russia.

Sites	Coordinates	Site area	Sample no.	Sediment unit	Dated material	Sample m a.s.l.	Lab no.	Conv. 14C age ( ± 1σ)	Cal. yr BP ( ± 1σ)	Context
*Bol'shaya*	N72° 32,384′	1	BBR 1:2	*	organic detritus	49.5	LuS_9344	8675 ± 60	9638 ± 88	fluvial/ice complex
*Balaknya River 1*	E100° 25,876′		BBR 1:3	*	organic detritus	48.8	LuS_9345	8175 ± 60	9130 ± 89	fluvial/ice complex
*Bol'shaya*	N73° 38,030′	2	BBR 2:1	unit B2	organic detritus	54.9	LuS_9346	>46,000	–	off-shore marine
*Balaknya River 2*	E100° 24, 914′		BBR 2:5	unit B1	mollusc fragments	53.9	LuS_9347	>48,000		off-shore marine
*Bol'shaya*	N73° 36,775′		BBR 4:3	unit A1	mollusc fragments	56.5	LuS_9348	>47,000	–	marine delta
*Balaknya River 4*	E100° 20,693′				(Astarte borealis)					
*Bol'shaya*	N73° 31,572′	3	BBR 6:1	unit A1	organic detritus	35.6	LuS_9349	>48,000	–	glaciomarine
*Balaknya River 6*	E101° 0,610′		BBR 6:3	unit A1	*Astarte borealis*	39.4	LuS_9350	>47,000	–	glaciomarine
			BBR 6:5	unit A1	wood (twig)	43.3	LuS_9351	>48,000	–	glaciomarine
			BBR 6:7	unit A1	organic detritus	45.4	LuS_9352	>48,000	–	glaciomarine
			BBR 6:10	unit A2	Macoma calcaria	49.5	LuS_12509	>48,000	–	glaciomarine
			BBR 6:11	unit A2	wood (twig)	48.9	LuS_9354	>48,000	–	glaciomarine
			BBR 6:17	unit B	mammoth tusk	56.5	LuS_12759	>48,000	–	fluvial
*Bol'shaya*	N73° 31,008′	3	BBR 7:1	*	wood, macrofossil	37.95	LuS_10135	7115 ± 55	7943 ± 54	fluvial/ice complex
*Balaknya River 7*	E101° 0,352′		BBR 7:2	*	macrofossil	38.05	LuS_ 10136	7190 ± 55	8005 ± 62	fluvial/ice complex
			BBR 7:3	*	wood (twig)	38.45	LuS_10137	7335 ± 55	8135 ± 76	fluvial/ice complex
			BBR 7:4	*	macrofossil	38.55	LuS_10138	5110 ± 55	5831 ± 68	fluvial/ice complex
			BBR 7:5	*	wood	39.05	LuS_10140	6690 ± 50	7560 ± 44	fluvial/ice complex
			BBR 7:6	*	macrofossil	39.95	LuS_10141	6720 ± 55	7587 ± 48	fluvial/ice complex
			BBR 7:7	*	macrofossil	40.50	LuS_10142	6500 ± 50	7414 ± 55	fluvial/ice complex
			BBR 7/TX029	*	mammoth (tusk)	35.0	LuS_13604	>42,000	–	redeposited beach finds close to section
			BBR 7/TX032	*	mammoth (scapula)	35.0	LuS_13605	33,800 ± 250	36,326 ± 359	
			BBR 7/TX035	*	mammoth (tusk)	36.0	LuS_13606	>48,000	–	
*Bol'shaya*	N73° 39,224′	4	BBR 8:3	unit A1	wood (twig)	40.5	LuS_9355	>48,000	–	marine
*Balaknya River 8*	E102° 10,223′		BBR 8:5	unit A1	*Macoma balthica*	43.1	LuS_9356	>47,000	–	marine
			BBR 8:11	unit A1	*Macoma bathica*	47.0	LuS_9357	>48,000	–	marine
			BBR 8:12	unit B	organic detritus	54.2	ЛУ-6679	7680 ± 100	8483 ± 103	ice complex
			BBR 8:13	unit B	organic detritus	59.3	ЛУ-6662	750 ± 50	691 ± 41	ice complex
*Bol'shaya*	N73° 38,887′	4	BBR 9:1	*	wood	51.6	LuS_10143	15,310 ± 85	18,578 ± 100	ice complex
*Balaknya River 9*	E102° 6,467′		BBR 9:2	*	wood	52.0	LuS_10144	14,640 ± 75	18,021 ± 107	ice complex
			BBR 9:3	*	wood	52.5	LuS_10145	13,620 ± 75	16,428 ± 136	ice complex
			BBR 9:4	*	wood	52.8	LuS_10146	4655 ± 50	5411 ± 74	ice complex
			BBR 9:5	*	wood	53.1	LuS_10147	13,940 ± 75	16,897 ± 148	ice complex
			BBR 9:6	*	wood	53.5	LuS_10148	13,810 ± 70	16,708 ± 145	ice complex
			BBR 9:7	*	wood	53.6	LuS_10149	13,960 ± 75	16,928 ± 149	ice complex
			BBR 9:9	*	wood	53.9	LuS_10150	13,160 ± 7	15,807 ± 128	ice complex
			BBR 9:10	*	wood	54.2	LuS_10151	12,460 ± 70	14,614 ± 217	ice complex
			BBR 9:11	*	wood	54.5	LuS_10152	12,310 ± 65	14,322 ± 174	ice complex
			BBR 9:12	*	wood	54.8	LuS_10153	9330 ± 65	11,397 ± 124	ice complex
			BBR 9:14	*	wood	55.4	LuS_10154	6250 ± 55	7464 ± 53	ice complex
*Bol'shaya*	N73° 38,887′	4	BBR 10:1	*	wood	52.7	LuS_10155	14370 ± 70	17514 ± 118	ice complex
*Balaknya River 10*	E102° 6,467′		BBR 10:2	*	wood	53.5	LuS_10156	13301 ± 75	15996 ± 121	ice complex
			BBR 10:3	*	wood	53.8	LuS_10157	13590 ± 75	16378 ± 133	ice complex
			BBR 10:4	*	wood	54.1	LuS_10158	13280 ± 70	15968 ± 125	ice complex
			BBR 10:5	*	wood	54.7	LuS_10159	12845 ± 65	15321 ± 123	ice complex
*Bol'shaya*	N73° 26,525′	5	BBR 11:1	unit C	peat	23.8	LuS_9358	>48,000	–	fluvial point bar
*Balaknya River 11*	E103° 26,609′		BBR 11:5	unit C	organic detritus	31.6	LuS_9359	15,370 ± 80	18,644 ± 89	fluvial point bar
*Bol'shaya*	N73° 26,747′	5	BBR 12:3	unit A	*Hiatella arctica*	26.5	LuS_9360	>48,000	–	marine
*Balaknya River 12*	E103° 26,307′									
*Bol'shaya*	N73° 29,873′	6	BBR 14:6	unit A2	wood	27.7	LuS_9362	>48000	–	shallow marine
*Balaknya River 14*	E104° 13,599′									
*Bol'shaya*	N73° 25,832′	6	BBR 15:2	unit D	*Astarte montagui*	22.0	LuS_9363	>48,000	–	glaciomarine
*Balaknya River 15*	E104° 21,352′		BBR 15:4	unit D	*Hiatella arctica*	20.1	LuS_9364	>48,000	–	glaciomarine
*Luktakh River 2*	N72°59,585′	9	LuR 2:1	unit A	*Hiatella arctica*	54.2	LuS 10377	>48000	–	glaciomarine
	E92°07,511′									
*Luktakh River 10*	N73° 09,387′	12	LuR 10:1	*	plant macrofossils	23.2	LuS 10963	180 ± 40	175 ± 89	aeolian
	E93° 24,429′		LuR 10:8	*	plant macrofossils	18.9	LuS 10964	3615 ± 45	3927 ± 67	fluvial point bar
*Logata River 1*	N73° 06,577′	14	LoR 1:1	unit A	*Hiatella arctica*	20.2	LuS 10377	>48,000	–	glaciomarine
	E96° 09,367′									
*Logata River 2*	N73° 03,773′	14	LoR 2:3	unit A	*Hiatella arctica*	16.8	LuS 10378	>48,000	–	glaciomarine
	E96° 20,492′		LoR 2:5	unit A	*Hiatella arctica*	21.8	LuS 10379	>45,000	–	glaciomarine
*Logata River 3a*	N73°21,015′	15	LoR 3a_2C	unit E	plant macrofossils	38.1	LuS 13903	45,000 ± 2000	47,994 ± 1275	ice complex, resedimented
	E96° 58,462′		LoR 3a:4	unit E	bison molar	34.7	LuS 10967	43,100 ± 2000	46,746 ± 1620	ice complex
			LoR 3a:3	unit E	plant macrofossils	34.7	LuS 10965	42,000 ± 2000	45,863 ± 1770	ice complex
			LoR 3a:2	unit E	plant macrofossils	34.2	LuS 10966	40,500 ± 1500	44,408 ± 1451	ice complex
			LoR 3a:1	unit C	shell, undiff	24.4	LuS 10386	>47,000	–	marine
*Logata River 3b*	N73° 20,723′ E97° 00,462′	15	LoR 3b:1	unit D	twig, 2–5 mm	31.9	LuS 10383	>48,000	–	fluvial point bar
			LoR 3b:2	unit D	twig, 2–4 mm	31.1	LuS 10384	>48,000	–	fluvial point bar
			LoR 3b:3	unit D	Salix, Dryas leaves	28.1	LuS 10385	>48,000	–	fluvial point bar
*Logata River 3c*	N73° 20,278′	15	LoR 3c:2	unit C	*Hiatella arctica*	25.3	LuS 10387	>46,500	–	marine
	E97° 01,290′									
*Logata River 3d*	N73° 19,956′	15	LoR 3d:1	unit D	Salix, Dryas leaves	34.1	LuS 10380	48,200–3000/+4000	–	fluvial point bar
	E97° 00,866′		LoR 3d:2	unit D	Salix leaves	33.6	LuS 10381	>48,000	–	fluvial point bar
			LoR 3d:3	unit D	plant det.	31.6	LuS 10382	>48,000	–	fluvial point bar
*Logata River 6*	N73° 19,139′	16	LoR 6:4	unit B	shell undiff	54.8	LuS 10388	>48,000	–	shell in till
	E97° 32,471′

**Table 6:** Electron Spin Resonance (ESR) ages on molluscs from stratigraphic sections at sites along the Bol'shaya Balaknya River, the Luktakh – Upper Taimyra – Logata river system and the Novorybnoye site (Fig. 1). More exact site locations are seen in Fig. 6 and Fig. 15 in Möller et al. [1], and stratigraphic positions of samples are indicated in sediment logs in Figs. 7, 8, 9, 12, 13, 16, 17, 18 and 20 in Möller et al. [1].

Site	Coordinates	Site area	Sample no.	Sediment unit	Lab no.	Dated mollusc	m a.s.l.	Uin (ppm)	U (ppm)	Th (ppm)	K (%)	DΣ (mGy/a)	Ps (Gy)	ESR-age (ka)	Context
*Bol'shaya*	N73° 31,572'	3	BBR 6:13	unit A2	435–061	*Macoma baltica*	51.0	0.18	1.04	5.56	1.75	1724	153.2	89.2 ± 7.6	glaciomarine
*Balaknya River 6*	E101° 0,610′														
*Bol'shaya*	N73° 39,224'	4	BBR 8:5	unit A1	436–061	*Macoma baltica*	43.1	0.10	1.08	5.14	1.75	1947	165.1	85.1 ± 7.3	marine
*Balaknya River 8*	E102° 10,223′		BBR 8:6	unit A1	437–061	*Macoma baltica*	43.4	0.18	0.90	4.50	1.81	1909	162.9	85.6 ± 7.3	marine
			BBR 8:9	unit A1	438–061	*Macoma baltica*	45.9	0.10	0.86	4.09	1.46	1701	133.7	79.0 ± 9.4	marine
*Bol'shaya*	N73° 27,236'	6	BBR 13:4	unit C	439–061	*Astarte borealis*	34.2	0.31	0.93	5.76	1.95	1751	739.0	430.0 ± 41.3	
*Balaknya River 13*	E104° 8,580′														
*Bol'shaya*	N73° 29,873'	6	BBR 14:3	unit A2	440–061	*Macoma baltica*	28.7	0.18	0.49	1.72	1.91	1924	155.0	80.8 ± 8.6	shallow marine
*Balaknya River 14*	E104° 13,599′		BBR 14:5	unit A2	441–061	*Macoma baltica*	29.4	0.10	0.13	1.57	1.89	1824	148.4	81.5 ± 7.0	shallow marine
*Bol'shaya*	N73° 25,832'	6	BBR 15:1	unit D	442-061B1)	*Macoma calcaria*	22.0	0.42	0.65	3.23	1.68	1677	386.8	228.0 ± 14.01)	glaciomarine
*Balaknya River 15*	E104° 21,352′				442-061A1)	*Astarte montagui*		0.10	0.65	3.23	1.68	1650	365.0		
			BBR 15:3	unit D	443–061	*Hiatella arctica*	20.2	0.65	0.89	4.18	1.65	1614	371.4	232.0 ± 19.10	glaciomarine
*Bol'shaya*	N73° 30.977'	6	BBR 16D:1	unit C	453–012	*Hiatella arctica*	35.2	0.24	0.79	6.39	1.87	1795	304.0	170.6 ± 14.5	glaciomarine
*Balaknya River 16A*	E104° 33,069′														
*Bol'shaya*	N73° 37,084'	7	BBR 17A:1	unit A	444–061	*Portlandia arctica*	8.4	0.22	0.72	5.76	1.74	1919	199.2	104.5 ± 8.9	marine
*Balaknya River 17A*	E105° 38,178′		BBR 17A:2	unit A	445–061	*Portlandia arctica*	7.9	0.14	0.74	6.45	1.76	1771	178.3	101.0 ± 8.7	marine
			BBR 17A:3	unit A	446–061	*Portlandia arctica*	12.4	0.16	0.64	5.53	1.63	1802	180.2	100.5 ± 12.0	marine
*Bol'shaya*	N73° 37,314'	7	BBR 17B:2a	unit B	447–061	*Portlandia arctica*	4.0	0.19	0.20	0.90	1.79	1761	214.3	122.3 ± 14.5	redeposited marine
*Balaknya River 17B*	E105° 39,092′		BBR 17B:2b	unit B	447-061-OS2)	*Portlandia arctica*	4.0	0.14	0.20	0.90	1.79	1753	214.3	123.0 ± 14.6	redeposited marine
*Novorybnoye 1*	N72° 49,742'	8	Nov 1c:4	unit B	461–033	*undif fragm*	12.9	1.70	1.35	6.06	1.93	741.0	1.93	311.7 ± 24.8	glaciomarine
	E105° 47,142'		Nov 1c:7	unit D	481–103	*undif fragm*	19.0	0.95	1.38	6.93	1.88	2101	421.0	202.0 ± 19.1	glaciomarine
*Novorybnoye 2*	N72° 49,650'	8	Nov 2:1	unit E2	466–033	*Hiatella arctica*	16.5	0.36	0.17	0.61	1.27	1153	153.5	131.0 ± 11.0	shoreface marine
	E105° 47,073′														
*Luktakh River 1-3*	N72°59.585’	9	LuR 2:1	unit A	465–033	*Hiatella arctica*	54.2	1.20	1.22	5.90	1.67	1577	112.7	71.7 ± 5.9	glaciomarine
	E92°07.511′		LuR3:1	unit A	482–103	*Hiatella arctica*	59.1	0.40	1.01	5.83	1.64	1376	110.3	80.5 ± 6.8	glaciomarine
			LuR3:2	unit A	483–103	*Hiatella arctica*	58.1	0.13	1.06	5.92	1.95	1857	160.6	86.8 ± 7.5	glaciomarine
*Luktakh River 4*	N72° 59,084'	9	LuR 4:2	unit A2	487–103	*Hiatella arctica*	56.7	0.18	0.87	3.89	1.70	1456	171.5	118.5 ± 10.1	shallow marine
	E92° 12,187′		LuR 4:5	unit A3	484–103	*Hiatella arctica*	58.5	0.57	0.45	2.56	1.78	1380	131.2	95.5 ± 8.0	shallow marine
*Luktakh River 5*	N73° 0,944’	9	LuR 5:1	Unit A	477–103	*Hiatella arctica*	58.6	2.46	1.34	4.55	1.48	1730	135.7	78.7 ± 6.2	glaciomarine
	E92°05,528′		LuR 5:2	unit A	479–103	*Hiatella arctica*	58.6	1.53	1.33	4.07	1.50	1569	126.3	80.8 ± 6.5	glaciomarine
*Luktakh River 6a*	N72° 51,161'	10	LuR 6a:5	unit C2	476–103	*Macoma baltica*	48.0	0.94	0.80	3.86	1.86	1917	149.0	78.0 ± 6.5	glaciomarine
	E92° 28,957′		LuR 6a:6	unit C2	486–103	*Macoma baltica*	45.8	0.42	1.42	4.9	1.79	1940	165.2	85.5 ± 7.3	glaciomarine
*Luktakh River 6b*	N72°51,132′		LuR 6b:1	unit C2	488–103	*Macoma ? (fragm)*	32.1	0.72	0.71	4.31	1.65	1652	141.5	86.0 ± 9.6	glaciomarine
	E92°28,797′		LuR 6b:2	unit C2	478–103	*Hiatella arctica*	31.3	0.45	1.34	4.73	1.69	1620	140.7	86.7 ± 7.3	glaciomarine
			LuR 6b: 5	unit C2	470–043	*Hiatella arctica*	29.8	0.35	1.08	4.1	1.44	1158	106.3	92.1 ± 7.8	glaciomarine
			LuR 6b:6	unit C1	471–043	*Hiatella arctica*	29.1	0.33	0.66	2.20	1.32	1151	94.3	82.2 ± 7.0	glaciomarine
*Luktakh River 8*	N72° 51,910'	11	LuR 8:1	unit A	462–033	*Hiatella arctica*	18.2	0.61	0.81	4.59	1.65	1377	120.2	87.3 ± 7.3	marine
	E93° 27,623′		LuR 8:2	unit A	485–103	*Hiatella arctica*	21.7	0.28	0.65	4.28	1.99	1161	108.0	93.4 ± 9.1	marine
*Luktakh River 9*	N72° 48,826′	11	LuR 9b:1	unit B	475–103	*Hiatella arctica*	43.7	0.57	0.91	3.17	1.17	1113	100.5	90.6 ± 7.5	beach-face marine
	E93° 22,093′		LuR 9b:2	unit B	472–103	*Hiatella arctica*	43.5	0.32	0.72	2.29	1.22	1139	103.8	91.5 ± 7.7	beach-face marine
*Logata River 1*	N73° 06,77’	14	LoR 1:1	unit A	463–033	*Hiatella arctica*	20.2	0.66	1.27	5.77	1.87	1980	206.1	104.5 ± 8.8	glaciomarine
	E96° 09,367′														
*Logata River 2*	N73° 03,773’	14	LoR 2:1	unit A	467–033	*Hiatella arctica*	27.6	0.31	1.25	5.37	1.9	1498	116.5	78.0 ± 6.6	glaciomarine
	E96° 20,492′		LoR 2:2	unit A	468–033	*Hiatella arctica*	26.4	0.40	1.28	4.93	1.70	1325	109.1	82.6 ± 7.0	glaciomarine
			LoR 2:4	unit A	469–033	*Hiatella arctica*	22.0	0.61	0.88	4.86	1.72	1300	121.4	93.7 ± 7.8	glaciomarine

All ESR dates were carried out by Dr. A. Molodkov at the Research Laboratory for Quaternary Geochronology, Institute of Geology, Tallin Technical University, Estonia.

Notes: U_in_ is the uranium content in shells; U, Th, K are the uranium, thorium and potassium content in sediments; D_Σ_ is the total dose rate; P_s_ is the palaeodose.

1) Two shells of different species from the same sample were analyzed, and mean age taken.

2) The sample was dated by the ESR open system (ESR-OS) method (Molodkov, 1988).

**Table 7:** Optically Stimulated Luminescence (OSL) ages from stratigraphic sections at sites along the Bol'shaya Balaknya River, the Luktakh – Upper Taimyra – Logata river system and the Novorybnoye site (Fig. 1). More exact site locations are seen on Fig. 6 and Fig. 15 in [1], and stratigraphic positions of samples are indicated in sediment logs in [1], Figs. 7, 8, 9, 12, 13, 14, 16, 17, 18, 19 and 20.

Site	Coordinates	Site	Samle code	Sediment unit	OSL lab. code	m a.s.l.	quartz OSL De Gy	n	age ratio IR50/OSL	age ratio pIRIR290/OSL	quartz OSL age, ka	prob. well reset	well reset	Context
Bol'shaya	N72° 32,384′	1	BBR 1:1a	no log	R-111003	420	35.0 ± 2	26	0.38 ± 0.04	1.10 ± 0.12	30 ± 2	✓	✓	fluvial/ice complex
Balaknya River 1	E100° 25,876′		BBR 1:1b	no log	R-121001	490	31.8 ± 1.1	31	0.68 ± 0.10	1.9 ± 0.4	16.5 ± 1.0	✓		fluvial/ice complex
Bol'shaya	N73° 38,030′	2	BBR 2:2	unit B2	R-111004	54.6	>250	40	<0.5	<1.2	>75	✓	✓	off-shore marine
Balaknya River 2	E100° 24, 914′		BBR 2:3	unit B1	R-111005	53.5	>250	21	<0.8	<1.6	>117	✓		off-shore marine
			BBR 2:4	unit B1	R-111006	52.4	>250	24	<0.7	<1.7	>104	✓		off-shore marine
Bol'shaya	N73° 36.775′	2	BBR 4:1	unit A1	R-111007	58.2	>250	38	<0.54	<0.98	>119	✓	✓	marine delta
Balaknya River 4	E100° 20.693′		BBR 4:2	unit A1	R-111008	57.8	202 ± 8	17	0.62 ± 0.06	1.5 ± 0.2	85 ± 5	✓		marine delta
Bol'shaya	N73° 31,572′	3	BBR 6:2	unit A	S-11077	37.2	>152	22	n/a	n/a	>49			glaciomarine
Balaknya River 6	E101° 0,610′		BBR 6:6	unit A	S-11078	43.7	264 ± 6	18	n/a	n/a	83 ± 6			glaciomarine
			BBR 6:8	unit A	R-121002	45.5	180 ± 9	35	0.63 ± 0.05	1.85 ± 0.16	92 ± 6	✓		glaciomarine
			BBR 6:9	unit A	R-121003	48.2	156 ± 11	36	0.85 ± 0.09	1.99 ± 0.19	77 ± 7	✓		glaciomarine
			BBR 6:14	unit B	S-11079	56.2	138 ± 3	24	n/a	n/a	50 ± 3			fluvial
			BBR 6:15	unit B	R-121004	57.0	88 ± 3	32	0.80 ± 0.09	1.38 ± 0.07	39 ± 2	✓		fluvial
Bol'shaya	N73° 39,224′	4	BBR 8:1	unit A1	S-11080	36.0	210 ± 3	24	n/a	n/a	97 ± 7			marine
Balaknya River 8	E102° 10,223′		BBR 8:2	unit A1	R-121005	39.5	156 ± 10	32	0.71 ± 0.07	1.40 ± 0.16	87 ± 6	✓		marine
			BBR 8:4	unit A1	S-11081	42.2	265 ± 10	18	n/a	n/a	96 ± 7			marine
			BBR 8:7	unit A1	R-121006	44.1	199 ± 12	36	0.67 ± 0.07	1.78 ± 0.15	89 ± 6	✓		marine
			BBR 8:10	unit A1	S-11082	48.0	275 ± 5	24	n/a	n/a	93 ± 6			marine
Bol'shaya	N73° 26,525′	5	BBR 11:2	unit C	R-111009	24.5	76 ± 3	30	0.58 ± 0.08	1.01 ± 0.12	46 ± 3	✓	✓	fluvial
Balaknya River 11	E103° 26,609′		BBR 11:3	unit C	R-121007	28.4	37.5 ± 1.4	26	0.37 ± 0.03	0.84 ± 0.07	19.3 ± 1.2	✓	✓	fluvial
			BBR 11:4	unit C	R-111010	30.7	42.1 ± 1.2	32	0.63 ± 0.08	1.27 ± 0.14	19.2 ± 1.0	✓	✓	fluvial
Bol'shaya	N73° 26,747′	5	BBR 12:1	unit A	R-111011	300	>250	21	<1.20	–	>131	−	−	marine
Balaknya River 12	E103° 26,307′		BBR 12:2	unit A	R-121008	150	>250	36	<1.7	–	>100	–	−	marine
Bol'shaya	N73° 27,584′	6	BBR13:1	unit A	R-111012	14.1	>250	32	<0.72	<1.7	>157	✓		fluvial
Balaknya River 13	E104° 9,881′		BBR13:2	unit A	R-121009	15.6	>250	18	<1.1	–	>124	✓		fluvial
			BBR13:3	unit A	R-121010	18.0	>250	18	<1.2	–	>118	✓		fluvial
			BBR13:5	unit D	R-111013	34.4	>250	20	<0.93	<2	>110	✓		shallow marine
			BBR13:6	unit D	R-121011	35.4	234 ± 20	18	0.99 ± 0.12	2.6 ± 0.3	119 ± 11	✓		shallow marine
			BBR13:7	unit D	R-121012	36.3	163 ± 12	17	0.87 ± 0.14	1.7 ± 0.3	100 ± 9	✓		shallow marine
Bol'shaya	N73° 29,873′	6	BBR 14:1	unit A1	R-111014	25.7	>250	29	<0.42	<0.9	>124	✓	✓	shallow marine
Balaknya River 14	E104° 13,599′		BBR 14:2	unit A2	R-111015	28.8	>250	21	<0.23	<0.6	>131	✓	✓	shallow marine
			BBR 14:4	unit A2	R-121016	30.0	216 ± 11	25	0.59 ± 0.05	1.03 ± 0.11	104 ± 7	✓	✓	shallow marine
Bol'shaya	N73° 25,832′	6	BBR 15:2	unit A	R-121015	11.0	>250	12	<0.9	<0.7	>120	✓		glaciotectonic def of ?
Balaknya River 15	E104° 21,352′		BBR 15:7	unit C	R-121014	15.0	>250	12	<0.9	<2	>167	✓		glaciomarine
			BBR 15:6	unit C	R-121013	16.0	>250	10	<0.9	<2	>131	✓		shallow marine
			BBR 15:5	unit C	R-111016	18.6	>250	19	<1.03	<2	>119	✓		shallow marine
			BBR 15:8	unit E	R-111017	22.7	80 ± 6	26	0.49 ± 0.05	0.7 ± 0.2	46 ± 4	✓	✓	aeolian
			BBR 15:1	unit E	R-111018	24.0	126 ± 6	26	0.57 ± 0.08	1.08 ± 0.15	57 ± 4	✓	✓	aeolian
Bol'shaya	N73° 30,964′	6	BBR 16A1:1	unit A	S-11072	12.5	>415	22	n/a	n/a	>138			shallow marine
Balaknya River 16A	E104° 32,033′		BBR 16A1:2	unit A	S-11073	15.0	>379	30	n/a	n/a	>121			shallow marine
			BBR 16A1:3	unit A	S-11074	18.8	>486	26	n/a	n/a	>163			shallow marine
			BBR 16A1:4	unit A	S-11075	20.8	>449	18	n/a	n/a	>153			shallow marine
			BBR 16A3:5	unit D	R-121017	38.8	127 ± 8	26	0.85 ± 0.20	1.21 ± 0.16	60 ± 5	✓	✓	aeolian
			BBR 16A3:6	unit D	S-11076	40.8	85.3 ± 1.2	26			32 ± 2			aeolian
Bol'shaya	N73° 31,004′	6	BBR 16C:1	unit A	R-111019	10.1	>250	15	<0.5	<1.4	>137	✓		shallow marine
Balaknya River 16C	E104° 32,621′		BBR 16C:2	unit A	R-121018	12.5	132 ± 10	21	1.30 ± 0.14	3.4 ± 0.5	100 ± 9	−	−	shallow marine
Bol'shaya	N73° 37,314′	7	BBR 17B:1	unit B	S-11083	8.0	103 ± 5	20	n/a	n/a	42 ± 4			fluvial
Balaknya River 17B	E105° 39,092′		BBR 17B:3	unit B	R-111020	9.0	71 ± 4	23	0.54 ± 0.06	1.16 ± 0.16	45 ± 3	✓	✓	fluvial
Novorybnoye 1a	N72° 49,742′ E105° 47,142′	8	Nov 1a:3	unit A	R-131001	11.0	>250	15	<1.9	–	>117	−	−	fluvial cretaceous
Novorybnoye 1c			Nov 1c:5	unit D	R-131002	15.0	>250	18	<0.9	<6	>129	✓		glaciomarine
			Nov 1c:6	unit D	R-131003	15.5	>250	27	<1.0	<5	>119	✓		glaciomarine
Novorybnoye 1e	N72° 49,771′ E105° 47,233′		Nov 1e:8	unit F	R-131004	26.5	26.3 ± 0.6	29	0.63 ± 0.05	1.17 ± 0.09	14.3 ± 0.7	✓	✓	aeolian
			Nov 1e:9	unit F	R-131005	27.0	26.5 ± 0.8	33	0.66 ± 0.05	0.97 ± 0.07	14.4 ± 0.8	✓	✓	aeolian
Novorybnoye 2	N72° 49,650′ E105° 47,073′	8	Nov 2:2	unit E2	R-131006	17.0	236 ± 16	18	0.56 ± 0.10	1.5 ± 0.3	124 ± 10	✓	✓	shallow marine
			Nov 2:3	unit E2	R-131007	16.0	>250	7	<0.8	<5	>182	✓		shallow marine
Novorybnoye 3	N72° 49,483′ E105° 47,002′	8	Nov 3:1	unit E2	R-131008	22.0	229 ± 12	15	0.70 ± 0.07	1.89 ± 0.30	101 ± 7	✓		shallow marine
			Nov 3:2	unit E2	R-131009	21.6	>250	28	<0.6	<5	>121	✓		shallow marine
Luktakh River 4	N72° 59,084′	9	LuR 4:3	unit A3	S-13002	57.8	240 ± 9	29	n/a	n/a	90 ± 6	✓	✓	shallow marine
	E92° 12,187′													
Luktakh River 6b	N72° 51,'1322′	10	LuR 6b:7	unit A	S-13007	24.7	381 ± 11	29	n/a	n/a	>144	✓	✓	marine?
	E92° 28,797′													
Luktakh River 8	N72° 51,910′	11	LuR 8:3	unit B	S-13009	23.4	87 ± 2	56	n/a	n/a	33 ± 2	✓	✓	fluvial
	E93° 27,623′		LuR 8:4	unit B	S-13010	25.9	82 ± 5114 ± 455	25	n/a	n/a	32 ± 3 43 ± 3			fluvial
Luktakh River 9	N72° 48,826′	11	LuR 9a:1	unit A	S-13011	39.1	279 ± 19	37	n/a	n/a	>84	✓	✓	glaciotectonic def of ?
	E93° 22,093′		LuR 9a:2	unit A	S-13012	38.6	306 ± 11	36	n/a	n/a	>99	✓	✓	glaciotectonic def of ?
Luktakh River 10	N73° 09,387′	12	LuR 10:1	no log	R-131017	23.7	0.16 ± 0.12	18	9.4 ± 1.5	60 ± 9	0.087 ± 0.012	(✓)	(✓)	aeolian
	E93° 24,429′		LuR 10:3	no log	R-131018	18.2	8.5 ± 0.2	19	0.68 ± 0.05	2.5 ± 0.2	4.7 ± 0.2	✓		fluvial point bar
			LuR 10:4	no log	R-131019	14.5	9.7 ± 0.3	17	0.68 ± 0.07	1.44 ± 0.11	5.2 ± 0.3	✓		fluvial point bar
Logata River 3b	N73° 20,723′	15	LoR 3b:4	unit D	R-131010	33.0	83 ± 4	24	0.83 ± 0.12	1.34 ± 0.19	48 ± 3	✓		fluvial point bar
	E97° 00,462′		LoR 3b:5	unit D	R-131013	30.0	105 ± 3	22	0.65 ± 0.08	1.08 ± 0.10	51 ± 3	✓	✓	fluvial point bar
			LoR 3b:6	unit D	R-131014	27.7	99 ± 2	24	0.56 ± 0.06	1.07 ± 0.19	57 ± 3	✓	✓	fluvial point bar
Logata River 3c	N73° 20,278′	15	LoR 3c:1	unit D	S-130101	29.1	61 ± 2	28	n/a	n/a	24.5 ± 1.7	✓	✓	fluvial
	E97° 01,290′													
Logata River 3d	N73° 19,956′	15	LoR 3d:5	unit D	R-131012	33.0	95 ± 5	27	0.59 ± 0.06	1.4 ± 0.2	50 ± 3	✓		fluvial point bar
	E97° 00,866′													
Logata River 6	N73° 19,139′	16	LoR 6:1	unit B	S-13004	100	227 ± 9	31	n/a	n/a	88 ± 6	✓	✓	Till boudin; marine sed?
	E97° 32,471′		LoR 6:2	unit A	S-13005	310	267 ± 8	26	n/a	n/a	>99	✓	✓	marine sed?
			LoR 6:3	unit A	S-13006	1010	158 ± 11,226 ± 10,335 ± 11	33	n/a	n/a	>61 > 87 > 128			marine sed?

**Table 8:** Properties and analytical data for boulders on the Sampesa (SA), Syntabul –Severokokorsky (NK) and Upper Taimyra – Baikuronyora (UT_B) Ice Marginal Zones (IMZ) analysed for cosmogenic^36^Cl (TCN exposure dating). Altitudes, latitudes, and longitudes were determined with GPS. For all samples, measured bulk rock density is 3.0 g/cm^3^, thickness is 5.0 cm, and topographic shielding is negligible. The rock dissolved indicates the amount processed for AgCl extraction chemistry. The Cl carrier is from PRIME Lab and has a^35^Cl/^37^Cl ratio of 273. Uncertainties on^35^Cl/^37^Cl and^36^Cl/Cl ratios and exposure ages represent propagated 1σ analytical/internal uncertainties only. Sample^36^Cl concentrations are corrected for^36^Cl contributed by procedural blanks. Exposure age uncertainties in parentheses incorporate external uncertainties, including production rate uncertainties; comparisons of the^36^Cl ages with those derived from independent chronometers (e.g., radiocarbon, OSL) must account for these external uncertainties. Ages “w/erosion” are calculated with a prescribed rock surface erosion rate of 1 mm/kyr. See Fig. 21 in [1] for site locations on map (*).

Sample	PRIME ID	Lat. (°N)	Lon. (°E)	Elev. (m)	Site # map*	Boulder size (m)	Rock diss. (g)	Cl carrier (mg)	^35^Cl/^37^Cl (±1σ)	^36^Cl/Cl (e^−15^, ±1σ)	^36^Cl conc. (e^4^ at/g, ±1σ)	Exposure Age (ka, ±1σ)	Age w/erosion (ka, ±1σ)
**Upper Taimyra – Baikuronyora IMZ**													
UT_B-1	201103318	73.96507	102.69740	134	1	0.7 × 0.7	31.1458	1.0550	5.951 ± 0.001	135.18 ± 9.45	17.73 ± 1.24	**22.1 ± 1.7 (2.4)**	**22.0 ± 1.6 (2.3)**
UT_B-2	201103319	73.79550	101.17040	123	2	2.6 × 2.0	30.3340	1.0406	3.437 ± 0.005	57.66 ± 8.18	39.96 ± 5.67	**15.6 ± 2.4 (3.5)**	**15.1 ± 2.2 (3.4)**
UT_B-3	–	73.99403	99.54113	163	3	2.3 × 1.6	–	–	–	–	–	–	–
UT_B-4	201900689	73.99402	99.54150	236	4	2.4 × 1.5	20.2981	1.0303	3.295 ± 0.021	79.30 ± 2.99	174.01 ± 21.97	**26.9 ± 4.1 (6.8)**	**25.2 ± 3.6 (6.1)**
**Syntabul – Severokokorsky IMZ**													
NK-1	201900690	73.98318	104.87208	130	5	2.0 × 2.0	20.1047	1.0281	6.285 ± 0.026	364.05 ± 8.95	72.32 ± 1.82	**84.1 ± 2.5 (7.8)**	**83.0 ± 2.9 (8.7)**
NK-2	201103320	73.96920	103.47693	137	6	1.5 × 1.3	31.4587	1.1095	3.666 ± 0.009	291.41 ± 10.69	146.35 ± 5.37	**81.0 ± 3.8 (13)**	**72.0 ± 3.9 (12)**
NK-3	–	73.96918	103.47695	143	7	1.2 × 1.2	–	–	–	–	–	–	–
NK-4	–	73.04255	101.33038	155	8	0.8 × 0.6	–	–	–	–	–	–	–
NK-5	201900691	73.04275	101.33102	156	9	0.7 × 0.6	20.1122	1.0276	4.740 ± 0.019	327.81 ± 6.82	100.57 ± 2.27	**79.5 ± 2.8 (9.9)**	**74.8 ± 2.8 (9.9)**
NK-6	–	73.73607	98.38002	189	10	1.7 × 1.5	–	–	–	–	–	–	–
NK-7	201900692	72.20930	101.63160	175	11	2.7 × 2.5	20.0517	1.0284	6.237 ± 0.034	499.23 ± 10.96	100.54 ± 2.31	**109 ± 3.1 (9.5)**	**110 ± 3.6 (11)**
NK-8	201103321	73.44448	102.80750	137	12	0.7 × 0.6	30.4033	1.0197	3.504 ± 0.200	281.91 ± 11.88	185.85 ± 7.83	**92.0 ± 4.6 (17)**	**82.0 ± 4.3 (15)**
**Sampesa IMZ**													
SA-1	201103322	72.01557	97.55150	131	13	1.0 × 0.9	30.7504	1.0578	7.276 ± 0.035	776.30 ± 28.52	89.30 ± 3.28	**131 ± 5.8 (11)**	**139 ± 7.1 (14)**
SA-2	201900693	72.01587	97.55788	121	14	0.8 × 0.8	20.1556	1.0283	3.351 ± 0.015	322.60 ± 7.77	541.72 ± 36.83	**120 ± 11 (29)**	**98.0 ± 8.4 (22)**
SA-3	201900694	72.20662	98.45890	65	15	0.7 × 0.7	20.2505	1.0264	10.145 ± 0.104	310.07 ± 8.30	41.81 ± 1.16	**54.5 ± 1.7 (3.8)**	**55.4 ± 1.8 (4.2)**
SA-4	201103323	72.20757	98.45793	78	16	0.7 × 0.7	3.6687	1.0757	7.308 ± 0.001	359.88 ± 13.12	346.22 ± 12.62	**249 ± 15 (51)**	**215 ± 15 (49)**
**Procedural blank**													
CLBLK-20	201900696	–	–	–	–	–	–	1.0285	167.1 ± 22.2	5.86 ± 0.90	–	–	–

Samples are sorted beneath their respective Ice Marginal Zones (IMZ), named in bold.

**Table 9:** Major element chemistry of boulder samples analysed for cosmogenic^36^Cl. All major element chemistry and LOI is listed in weight percent and was performed with XRF with 0.01% detection limit. H_2_O and CO_2_ are each assumed to account for half the LOI signal.

Sample	SiO_2_	TiO_2_	Al_2_O_3_	Fe_2_O_3_	MnO	MgO	CaO	Na_2_O	K_2_O	P_2_O_5_	Cr_2_O_3_	LOI
UT_B-1	48.90	0.74	14.50	11.50	0.18	10.50	10.90	2.15	0.42	0.08	0.08	0.12
UT_B-2	49.00	0.92	14.60	11.30	0.18	9.06	11.60	2.18	0.46	0.09	0.06	0.59
UT_B-4	53.40	2.62	14.00	12.30	0.18	3.94	6.95	3.89	2.25	0.15	0.01	0.00
NK-1	50.00	0.80	15.70	9.87	0.17	8.52	11.20	2.39	0.60	0.03	0.06	0.28
NK-2	51.60	0.81	14.60	10.20	0.18	7.43	10.90	2.19	1.01	0.10	0.03	1.16
NK-5	51.00	0.94	14.10	11.10	0.18	7.81	12.00	2.08	0.75	0.03	0.01	0.03
NK-7	52.80	2.73	14.00	12.80	0.19	4.13	7.16	3.84	2.17	0.04	0.02	0.00
NK-8	50.90	0.94	14.20	10.90	0.18	7.68	10.80	2.31	0.88	0.12	0.07	0.78
SA-1	49.50	0.93	15.00	11.30	0.18	9.03	11.20	2.21	0.52	0.10	0.07	0.15
SA-2	51.80	0.95	13.90	10.70	0.18	6.98	11.50	2.24	0.91	0.02	0.01	0.90
SA-3	51.80	0.94	14.00	11.00	0.18	7.22	11.70	2.22	0.87	0.02	0.01	0.04
SA-4	52.20	0.86	14.00	11.60	0.18	7.83	10.40	2.08	1.00	0.10	0.04	0.28

**Table 10:** Trace element chemistry of boulder samples analysed for cosmogenic^36^Cl, expressed in ppm. Cl is calculated using isotope dilution based on AMS data from PRIME Lab. Trace elements were analysed by ICP-OES with detection limits (ppm) as follows: 10 for B, Cr, Li; 0.1 for Sm, Th; 0.05 for Gd, U.

Sample	Cl (±1σ)	B	Sm	Gd	U	Th	Cr	Li
UT_B-1	48.9 ± 4.2	<10	2.0	2.53	0.17	0.7	521	<10
UT_B-2	458.7 ± 92.3	<10	2.5	3.07	0.18	0.8	419	<10
UT_B-4	1243.6 ± 155.6	<10	5.4	5.29	1.31	4.6	51	15
NK-1	65.4 ± 0.6	<10	1.7	2.02	0.22	0.8	395	<10
NK-2	270.3 ± 11.3	<10	3.6	3.52	0.69	2.7	188	15
NK-5	129.2 ± 1.5	<10	2.4	2.92	0.59	1.8	63	<10
NK-7	66.7 ± 0.8	<10	4.6	4.70	1.31	4.2	53	12
NK-8	368.2 ± 17.4	<10	3.5	3.79	0.47	2.0	422	17
SA-1	33.6 ± 1.3	<10	2.6	3.09	0.26	1.1	441	<10
SA-2	937.2 ± 62.8	<10	2.5	2.76	0.69	2.0	46	11
SA-3	28.5 ± 0.5	<10	2.8	3.22	0.65	2.0	47	11
SA-4	284.2 ± 11.4	<10	3.9	4.21	0.82	3.1	236	12

### 2.3 Marine mollusc faunas

Molluscs were collected during stratigraphic work, both for dating purposes (^14^C, ESR) and, when encountered in larger numbers, for determination of the marine mollusc fauna for the relevant stratigraphic units (Table 3). The analyses were carried out at the Geological Museum, University of Copenhagen, Denmark. The biostratigraphy of Siberian raised marine sediments based on mollusc faunas has traditionally played an important role in the construction of a Pleistocene stratigraphy and reconstruction of palaeoenvironments, based on the species’ present distribution, e.g. [9]. The species are classified according to their present distribution into *Subarctic* (SA), *Arctic* (A), and *non-indicative* (N/A). This is based on oceanographical parameters, notably the inflow of Atlantic water into the Arctic, a decisive factor in the distribution of near-shore marine ecosystems, and absence/duration of sea ice [10]. Subarctic species occur in the zone where Atlantic and Arctic water masses mix and seasonal sea ice occurs, such as today in the southern and eastern Barents Sea and western part of the Kara Sea, while Arctic species thrive in Arctic water masses with long lasting sea ice cover. A third biogeographical group, the Boreal species, is restricted to permanently ice free coasts. None of these species have been observed in the present material, although they occur in interglacial sediments in the Yenissei River basin to the south [9]. At present the eastern Kara Sea is dominated by Arctic water masses, but with a high inflow of fresh river water in the southern part [11].

### 2.4 Terrestrial and limnic macrofossil analyses

Organic debris in fluvial ripple-laminated successions was analysed from one site (LoR 3, Fig. 1), five samples in total, for their macrofossil content (Table 4). The samples were wet-sieved (mesh ≥0.1 mm) and the residue left on the sieves was analysed using a Leica Wild dissecting microscope (analysed at Geological Survey of Denmark and Greenland (GEUS), Denmark (macrofossils)). The plant names are according to http://www.theplantlist.org/. Leaves, seeds and fruits were well preserved and come from local sources. The plant residue includes numerous remains of mosses; a few tentative identifications are included, but most moss remains were not identified. The remains of mosses usually preserve well and often dominate Quaternary macro-floras from the Arctic, reflecting that mosses are important constituents of Arctic plant communities. Some animal remains, especially *Coleoptera* fragments, were also identified to genera or species level (analysed at the Dept. of Biology and Environmental Science, Linnaueus University, Sweden (insects))

### 2.5 Geochronology

Four dating methods were employed: Accelerator Mass Spectrometer radiocarbon dating (AMS ^14^C; molluscs, terrestrial organic material), Electron Spin Resonance (ESR; molluscs), Optically Stimulated Luminescence (OSL; sediment) and *in situ* Terrestrial Cosmogenic Nuclide surface exposure dating (TCN; boulders).

*Radiocarbon dating.* – A total of 66 AMS ^14^C ages were determined at the AMS Radiocarbon Dating Laboratory, Department of Geology at Lund University, Sweden (Table 5). Pre-treatment of mollusc shells included leaching to ∼70% of their original mass. Finite ages from terrestrial material (wood, organic detritus, plant macrofossils, bone) are given as conventional radiocarbon years (^14^C age BP) with 1σ age deviation, as well as calibrated calendar years (cal yr BP or cal ka BP), calculated with the software package Oxcal 4.3.2 [12] and with use of IntCal 13 (mean age ±1σ).

*ESR dating*. – A total of 39 marine mollusc samples were dated by Electron Spin Resonance (ESR) at the Research Laboratory for Quaternary Geochronology at Tallinn Technical University, Estonia (Anatoly Molodkov) (Table 6). Unexposed shells were retrieved from within cleaned sections, followed by sampling of sediments enclosing the sampled shell for later measurements of background dose rates. The method is based on direct measurements of the amount of radiation-induced paramagnetic centres, trapped in the fossil shell substance and created by the natural radiation resulting from radioactivity in the shell itself and from the enclosing sediment. Standard analytical procedures were used according to Molodkov [13] and Molodkov et al. [14] and ESR age were calculated from the measured total radiation dose that the shell received during its burial versus dose rate [15]. In some sediment sections where sediment logs indicate the presence of molluscs it was unfortunately not possible to retrieve molluscs for ESR dating, either because they were too low in concentration, very friable and/or partly dissolved *in situ*. Although their presence was confirmed by weathered-out and hardened shells lying on exposed sediment surfaces, such shells are un-suitable for ESR dating because of prolonged daylight exposure and the difficulty of unambiguous identification of samples of the relevant burial sediment.

*OSL dating.* – A total of 76 sediment samples were dated by Optically Stimulated Luminescence (OSL) (Table 7). Sediment samples were taken by means of hammering 20 cm long PVC tubes into cleaned pit walls of suitable sediment (see Fig. 5C in [1]). Samples marked with an OSL laboratory code R-xxxxxx (Table 7) were processed at Aarhus University's Nordic Laboratory for Luminescence (NLL) Dating located at the Risø Campus, Roskilde, Denmark, while samples marked S-xxxxx were handled at SCIDR Luminescence Laboratory, Sheffield University, UK. After conventional grain-size and density separation and subsequent chemical purification, the single aliquot regenerative (SAR) dose protocol was applied to multi-grain (180–250 μm) quartz aliquots (8 mm diameter, typically >18 per sample) to estimate the equivalent dose, D_e_ [16], [17]), using blue (470 ± 30 nm) light stimulation, 260 °C preheating for 10 s, and a cut heat of 220 °C. Photon detection was through a U-340 glass filter, and the signal used for D_e_ determination was based on the first 0.8 s of OSL, less a background based on the signal detected between 1.6 and 2.4 s of stimulation. To test the applicability of this chosen protocol to the measurement of the dose recoded by the quartz OSL signal, we applied a dose recovery test ([18]) to at least 3 aliquots from each sample dated at the NLL, after initial bleaching with blue light for 100s, followed by a 10 ks pause and a further 100s bleach. The average measured/given dose ratio is 0.999 ± 0.011 (n = 168) demonstrating that our protocol is able to accurately measure a dose given to a sample prior to any laboratory heating. The equivalent doses (D_e_), measured for each sample are given in Table 7.

Because feldspar infra-red stimulated luminescence (IRSL) signals are more difficult to reset by daylight than the OSL signals from quartz [19], [20], the apparent quartz and feldspar deposition ages of a particular sediment give information on the probability that the most light sensitive signal (quartz OSL) was fully reset prior to deposition. Accordingly, multi-grain (180–250 μm) feldspar aliquots (3 mm diameter, at least 3 aliquots per sample) extracted from the samples processed by NLL were measured using a post IR-IR SAR protocol, with a preheat temperature of 250 °C for 1 minute, and stimulation with IR (870 nm) for 100 s while the aliquot was held at 50 °C (IR_50_), followed by a further 100 s with the sample held at 225 °C (pIRIR_225_) [21] ( [22]. Detection was through BG-39 and 7–59 filters. Signals used for dose estimation were based on the first 4 s of stimulation, less a background based on the signal between 95 and 100 s of stimulation. Multi-grain quartz and feldspar aliquots were employed because this study aims to identify well-bleached samples; the average dose is then the most appropriate dose estimate [23], and for a given number of measurements, this is most precisely measured using large aliquots.

The samples were analysed for natural radionuclide concentrations in the laboratory, using high-resolution gamma spectrometry [24], [25]. These concentrations were converted into dose rates using conversion factors listed by Olley [26]; a cosmic ray contribution was calculated according to [27], assuming the modern burial depth has applied throughout the lifetime of the site. Both field and laboratory saturated water contents were measured. The resulting total dose rates to quartz are summarised in Table 7; the dose rates to feldspar can be derived by adding 0.81 Gy/ka to these values (based on an assumed concentration of 12 %K in feldspar extracts [28].

The quartz ages resulting from the measurements described above are summarised in Table 7, together with the ratios of the feldspar IR_50_ and pIRIR_225_ ages to quartz OSL ages (for the NLL-measured samples). The quartz ages are then characterised as ‘probably well bleached’, ‘well bleached’ or unknown based on these age ratios, following Möller and Murray [29].

*Terrestrial Cosmogenic Nuclide (TCN) (*^*36*^*Cl) exposure dating.* – Erratic boulders on top of the major ice-marginal zone ridges were scouted by means of Mi8 helicopter transport, with flights over the ridges at 150 km/hr at 100 m height. We flew for a total of 2 days and covered ∼1500 km in total distance, but large boulders suitable for ^36^Cl exposure dating proved difficult to find. Unfortunately, the Urdakh IMZ (‘U’ on Fig. 1) is covered with a sparse larch forest, and this prevented landing at potentially suitable boulders. Sampling was, however, possible at 11 sites along the Sampesa, the Syntabul – Severokokorsky and the Upper Taimyra – Baikuronyora ice marginal zones (Fig. 1), and with double sampling at a few sites, 16 boulders were sampled in total.

Samples were collected from the top surface of the largest available boulders in the vicinity, using an angle grinder and sawing the boulder in a cross-hatched pattern(see Fig 5D and E in [1]), enabling an exact estimate of the sample thickness. All sampled boulders were basalt and rested on flat surfaces on the crest of the IMZs. Sample coordinates and altitudes were obtained in the field using a handheld GPS. Topographic shielding was negligible for all sampled boulders. The dry bulk density was measured before crushing and sieving to the 250-125 μm fraction at Lund University, and averaged 3.0 g/cm^3^ (Table 8). From each sample, c. 10 g was retained for whole rock elemental analyses at SGS Minerals Services, Canada, where major and trace elements were measured using X-ray fluorescence (XRF) and inductively coupled plasma – optical emission spectrometry (ICP-OES), respectively (Table 9, Table 10).

Six samples (UT_B-1, UT_B-2, NK-2, NK-8, SA-1, SA-4) were chemically prepared at PRIME Lab, Purdue University, USA, for AMS measurement following standard protocols at this facility. Chemical preparation of the remaining six samples (UT_B-4, NK-1, NK-5, NK-7, SA-2, SA-3) was performed in the Cosmogenic Isotope Clean Lab at the University of New Hampshire, USA, following methods developed by Stone et al. [30] and modified by Licciardi et al. [31]. Milled samples were ultrasonically cleaned in deionized water, pre-treated with 2% HNO_3_, and spiked with an enriched ^35^Cl tracer supplied by PRIME Lab, then dissolved in HF–HNO_3_ solution. Upon complete digestion, insoluble fluoride compounds were removed by centrifuging and Cl was precipitated as AgCl with the addition of AgNO_3_. The precipitate was further purified by re-dissolution in NH_4_OH and the addition of BaNO_3_ to precipitate sulphate as BaSO_4_. AgCl was then re-precipitated by addition of 2M HNO_3_ and AgNO_3_, washed repeatedly in deionized water, and dried in an oven.

All ^35^Cl/^37^Cl and ^36^Cl/Cl ratios were measured at the PRIME Lab facility. Appropriate corrections for a procedural blank (CLBLK-20) were made prior to age calculations and accounted for 0.1–1.6% adjustments to the ^36^Cl concentrations in the unknowns. Ages were calculated with the online CRONUScalc ^36^Cl exposure age calculator using the LSDn scaling scheme [32], [33], [34]. Sensitivity analyses were conducted using the CRONUScalc calculator [33], [34] to evaluate the potential impact of a rock surface erosion rate of 1 mm/kyr on the apparent exposure ages (Table 8).

## References

[1] Möller P., Benediktsson Í.Ö., Anjar J., Bennike O., Bernhardson M., Funder S., Håkansson L., Lemdahl G., Licciardi J.M., Murray A.S., Seidenkrantz M.-S. (2019). Glacial history and palaeo-environmental change of southern Taimyr Peninsula, arctic Russia, during the middle and late Pleistocene. Earth Sci. Rev..

[2] Möller P., Alexanderson H., Funder S., Hjort C. (2015). The Taimyr Peninsula and the Severnaya Zemlya archipelago, Arctic Russia: a synthesis of glacial history and palaeo-environment change during the last glacial cycle (MIS 5e-2). Quat. Sci. Rev..

[3] V Kind N., Leonov B.N. (1982). Antropogen Taimyra (The Antropogen of the Taimyr Peninsula).

[4] Möller P., Hjort H., Alexanderson H., Sallaba F., Ehlers J., Gibbard P.L., Hughes P.H. (2011). Glaciation history of the Taymyr Peninsula and the Severnaya Zemlya archipelago, Arctic Russia. Quaternary Glaciations - Extent and Chronology - a closer look.

[5] Alexanderson H., Hjort C., Möller P., Antonov O., Pavlov M. (2001). The North Taymyr ice-marginal zone, Arctic Siberia - a preliminary overview and dating,. Glob. Planet. Chang..

[6] Jakobsson M., Mayer L., Coakley B., Dowdeswell J.A., Forbes S., Fridman B., Hodnesdal H., Noormets R., Pedersen R., Rebesco M., Schenke H.W., Zarayskaya Y., Accettella D., Armstrong A., Anderson R.M., Bienhoff P., Camerlenghi A., Church I., Edwards M., Gardner J.V., Hall J.K., Hell B., Hestvik O., Kristoffersen Y., Marcussen C., Mohammad R., Mosher D., Nghiem S.V., Pedrosa M.T., Travaglini P.G., Weatherall P. (2012). The international bathymetric Chart of the Arctic Ocean (IBCAO) version 3.0. Geophys. Res. Lett..

[7] Eyles N., Eyles C.H., Miall A.D. (1983). Lithofacies types and vertical profile models; an alternative approach to the description and environmental interpretation of glacial diamict and diamictite sequences. Sedimentology.

[8] Feyling-Hanssen R.W., Costa L.I. (1983). Quantitative methods in micropaleontology.

[9] Troitsky S.L. (1966). Quaternary Deposits and Relief of the Low Coastlands of the Yenisei Estuary and Adjacent Byrranga Mountains (In Russian).

[10] Funder S., Demidov I., Yelovicheva, Y Y. (2002). Hydrography and mollusc faunas of the baltic and the white-north sea seaway in the eemian. Palaeogeography, Palaeoclimatology, Palaeoecology.

[11] Kulakov M.Y., Pogrebov V.B., Timofeyev S.F., Chernova N.V., Kiyko O.A., Robinson A.R., Brink K.H. (2004). Ecosystem of the Barents and Kara seas, coastal segment. The Sea, Volume 14B: the Global Coastal Ocean.

[12] Bronk Ramsey C. (2017). Methods for summarizing radiocarbon data sets. Radiocarbon.

[13] Molodkov A.N. (1988). ESR dating of Quaternary shells: recent advances. Quat. Sci. Rev..

[14] Molodkov A.N., Dreimanis A., Ăboltiņš O., Raukas A. (1988). The ESR age of *Portlandia arctica* shells from glacial deposits of Central Latvia: an answer to a controversy on the age and genesis of their enclosing sediments. Quat. Sci. Rev..

[15] Molodkov A.N., Bolikhovskaya N.S. (2002). Eustatic sea-level and climate changes over the last 600 ka as derived from mollusc-based ESR-chronostratigraphy and pollen evidence in Northern Eurasia. Sediment. Geol..

[16] Murray A.S., Wintle A.G. (2000). Luminescence dating of quartz using an improved single-aliquot regenerative-dose protocol. Radiat. Meas..

[17] Murray A.S., Wintle A.G. (2003). The single aliquot regenerative dose protocol: potential for improvements in reliability. Radiocarbon Measurements.

[18] Murray A.S. (1996). Incomplete stimulation of luminescence in young quartz sediments and its effect on the regenerated signal. Radiat. Meas..

[19] Godfrey-Smith D.L., Huntley D.J., Chen W.H. (1988). Optically dating studies of quartz and feldspar sediment extracts. Quat. Sci. Rev..

[20] Murray A.S., Thomsen K.J., Masuda N., Buylaert J.P., Jain M. (2012). Identifying well-bleached quartz using the different bleaching rates of quartz and feldspar luminescence signals. Radiat. Meas..

[21] Thomsen K.J., Murray A.S., Jain M., Bøtter-Jensen L. (2008). Laboratory fading rates of various luminescence signals from feldspar-rich sediment extracts. Radiat. Meas..

[22] Buylaert J.P., Murray A.S., Thomsen K.J., Jain M. (2009). Testing the potential of an elevated temperature IRSL signal from K-feldspar. Radiat. Meas..

[23] Guerin G., Jain M., Thomsen K.J., Murray A.S., Mercier N. (2015). Modelling dose rate to single grains of quartz in well-sorted sand samples: the dispersion arising from the presence of potassium feldspars and implications for single grain OSL dating. Quat. Geochronol..

[24] Murray A.S., Marten R., Johnston P., Martin A.J. (1987). Analysis for naturally occurring radionuclides at environmental concentrations by gamma spectrometry. J. Radioanal. Nucl. Chem..

[25] Murray A.S., Helsted L.M., Autzen M., Jain M., Buylaert J.P. (2018). Measurement of natural radioactivity: calibration and performance of a high-resolution gamma spectrometry facility. Radiat. Meas..

[26] Olley J.M., Murray A.S., Roberts R.G. (1996). The effects of disequilibria in uranium and thorium decay chains on burial dose rates in fluvial sediments. Quat. Geochronol..

[27] Prescott J.R., Hutton J.T. (1994). Cosmic-ray contributions to dose-rates for luminescence and ESR dating - large depths and long-term variations. Radiat. Meas..

[28] Huntley D.J., Baril M.R. (1997). The K content of the K-feldspars being measured in optical dating or in thermoluminescence dating. Ancient TL.

[29] Möller P., Murray A.S. (2015). Drumlinised glaciofluvial and glaciolacustrine sediments on the Småland peneplain, South Sweden – new evidence on the growth and decay history of the Fennoscandian Ice Sheets during MIS 3. Quat. Sci. Rev..

[30] Stone J.O., Fifield L.K., Allan G.L., Cresswell R.G. (1996). Cosmogenic chlorine-36 from calcium spallation. Geochem. Cosmochim. Acta.

[31] Licciardi J.M., Denoncourt C.L., Finkel R.C. (2008). Cosmogenic ^36^Cl production rates from Ca spallation in Iceland. Earth Planet. Sci. Lett..

[32] Lifton N., Sato T., J Dunai T. (2014). Scaling *in situ* cosmogenic nuclide production rates using analytical approximations to atmospheric cosmic-ray fluxes. Earth Planet. Sci. Lett..

[33] Marrero S.M., Phillips F.M., Borchers B., Lifton N., Aumer R., Balco G. (2016). Cosmogenic nuclide systematics and the CRONUScalc program. Quat. Geochronol..

[34] Marrero S.M., Phillips F.M., Caffee M.W., Gosse J.C. (2016). CRONUS-Earth cosmogenic ^36^Cl calibration. Quat. Geochronol..

